# *In Silico* Identification and Experimental Validation of Insertion–Deletion Polymorphisms in Tomato Genome

**DOI:** 10.1093/dnares/dsu008

**Published:** 2014-03-11

**Authors:** Jingjing Yang, Yuanyuan Wang, Huolin Shen, Wencai Yang

**Affiliations:** Beijing Key Laboratory of Growth and Developmental Regulation for Protected Vegetable Crops, Department of Vegetable Science, China Agricultural University, No. 2 Yuanmingyuan Xilu, Beijing 100193, China

**Keywords:** insertion–deletion, *Solanum lycopersicum*, genetic variation

## Abstract

Comparative analysis of the genome sequences of *Solanum lycopersicum* variety Heinz 1706 and *S. pimpinellifolium* accession LA 1589 using MUGSY software identified 145 695 insertion–deletion (InDel) polymorphisms. A selected set of 3029 candidate InDels (≥2 bp) across the entire tomato genome were subjected to PCR validation, and 82.4% could be verified. Of 2272 polymorphic InDels between LA 1589 and Heinz 1706, 61.6, 45.2, and 31.6% were polymorphic in 8 accessions of *S. pimpinellifolium*, 4 accessions of *S. lycopersicum* var. *cerasiforme*, and 10 varieties of *S. lycopersicum*, respectively. Genetic distance was 0.216 in *S. pimpinellifolium*, 0.202 in *S. lycopersicum* var. *cerasiforme*, and 0.108 in *S. lycopersicum*. The data suggested a reduction of genetic variation from *S. pimpinellifolium* to *S. lycopersicum* var. *cerasiforme* and *S. lycopersicum*. Cluster analysis showed that the 8 accessions of *S. pimpinellifolium* were in one group, whereas 4 accessions of *S. lycopersicum* var. *cerasiforme* and 10 varieties of *S. lycopersicum* were in the same group.

## 1. Introduction

Tomato (*Solanum lycopersicum* L.) is an economically important vegetable crop worldwide and a pre-eminent plant genetic analysis system. Genetic marker development for tomato has been conducted over 30 years through various approaches, including restriction fragment length polymorphism (RFLP), random ampliﬁed polymorphic DNA (RAPD), ampliﬁed fragment length polymorphisms (AFLPs), simple sequence repeat (SSR), cleaved ampliﬁed polymorphisms (CAPs), and conserved ortholog sets (COSs). Most markers developed by these approaches are based on DNA or cDNA polymorphisms between wild species and cultivated tomato, which lead to the construction of the first generation reference linkage maps and isolation of genes of interests.^1,2^ However, the ability of using these markers to detect polymorphisms in cultivated tomato is limited.^3^ Recent efforts to develop new markers in cultivated tomato have been focus on single-nucleotide polymorphisms (SNPs) using *in silico* mining of expressed sequence tag database and experimental validation,^4–7^ amplicon sequencing of COS genes,^8,9^ hybridization to oligonucleotide array,^10^ and next-generation sequencing of transcriptome or re-sequencing of genome.^11–13^ Owing to the abundance and wide distribution of SNPs in the whole genome and the availability of automatic large-scale genotyping platform, SNPs have widely been used in association analysis,^13–15^ high-density SNP map construction,^7,16^ as well as population structure and genetic variation analysis^17–20^ in cultivated tomato.

Short insertion and deletion (InDel) polymorphisms are increasingly being received attention in human because they are the second abundant form of genetic variation and can influence multiple human phenotypes including diseases.^21–25^ Therefore, great efforts have been put on identification, mapping, and functional analysis of InDels in the human genome.^26–28^ Similar work has been done in other species, such as Arabidopsis and rice.^29–33^ In tomato, a total of 749 966 putative InDels of 3–300 bp have been identified by comparing the genome sequences of *Solanum pimpinellifolium* accession LA 1589 and *S. lycopersicum* variety Heinz 1706,^34^ and more than 80 000 putative InDels of 1–15 bp have been discovered by comparative analysis of transcriptome between wild species *S. galapagense* and cultivated tomato.^35^ However, less work on discovery of InDels in cultivated tomato has been done.

The availability of the whole genome or transcriptome sequences provides a potential to identify InDels *in silico*. We here developed a pipeline to identify InDels by comparative analysis of the two available genome sequences of LA 1589 and Heinz 1706. A total of 3029 candidate InDels were subjected to experimental validation by PCR amplification of genomic DNA in a collection of 22 tomato lines. The main objective of this study was to develop easy-using markers for genetic study and marker-assisted selection in cultivated tomato.

## 2. Materials and methods

### 2.1. Plant materials and DNA isolation

A panel of 22 tomato genotypes comprising of cultivated tomato (*S. lycopersicum*) and its wild relatives were used to validate InDel polymorphisms. These inbred lines were selected to represent a diverse collection including eight accessions of *S. pimpinellifolium*, five processing varieties, one greenhouse cultivar, four fresh market cultivars, and four *S. lycopersicum* var. *cerasiforme* accessions (Table 1). Nine of them were used for SNP detection in our previous study.^9^ The eight *S. pimpinellifolium* accessions were selected from the core collection or sources being used for genetic studies and were used to detect polymorphisms of candidate InDels within the species. Genomic DNA was isolated from fresh-collected young leaves of at least eight plants for each genotype using the modified CTAB method.^36^

**Table 1.** Description of plant materials

Genotype	Species	Market type	Origin	Note
LA 1269	*Solanum pimpinellifolium*	Wild	Peru	Resistance source for late blight (*Ph-3*)
LA 1589	*Solanum pimpinellifolium*	Wild	Peru	Genome sequenced, widely used for genetic studies
PI 128216	*Solanum pimpinellifolium*	Wild	Bolivia	Resistance source for bacterial spot and bacterial speck
LA 0373	*Solanum pimpinellifolium*	Wild	Peru	Core collection
LA 0400	*Solanum pimpinellifolium*	Wild	Peru	Core collection
LA 0722	*Solanum pimpinellifolium*	Wild	Peru	Core collection
LA 1582	*Solanum pimpinellifolium*	Wild	Peru	Core collection
LA 2181	*Solanum pimpinellifolium*	Wild	Peru	Core collection
Heinz 1706	*Solanum lycopersicum*	Processing	USA	Genome sequenced
OH 88119	*Solanum lycopersicum*	Processing	USA	Early fruit set
OH 9242	*Solanum lycopersicum*	Processing	USA	High lycopene
Liger 87-5	*Solanum lycopersicum*	Processing	China	Current major variety in China
M 82	*Solanum lycopersicum*	Processing	Israel	Widely used in genetic studies
Money maker	*Solanum lycopersicum*	Greenhouse	USA	Widely used in genetic studies
Fla.7600	*Solanum lycopersicum*	Fresh market	USA	Variety with multiple disease resistance genes
Baiguoqiangfeng	*Solanum lycopersicum*	Fresh market	China	Previous major variety in China
Shijifeng	*Solanum lycopersicum*	Fresh market	China	Previous major variety in China
Zhongshu 5	*Solanum lycopersicum*	Fresh market	China	Previous major variety in China
Black cherry	*Solanum lycopersicum* var. *cerasiforme*	Cherry	USA	Brown fruit
LA 1310	*Solanum lycopersicum* var. *cerasiforme*	Cherry	Peru	Salt tolerance
LA 4133	*Solanum lycopersicum* var. *cerasiforme*	Cherry	USA	Core collection, salt tolerance
PI 114490	*Solanum lycopersicum* var. *cerasiforme*	Cherry	UK	Yellow fruit, resistance to bacterial spot

### 2.2. Prediction of InDels between LA 1589 and Heinz 1706

The genomic DNA sequences of *S. pimpinellifolium* accession LA 1589 (Spimpinellifolium_genome.contigs.fasta.gz) and *S. lycopersicum* variety Heinz 1706 (S_lycopersicum_chromosomes.2.40.fa.gz) were downloaded to a local computer from the SOL Genomics Network (SGN, http://solgenomics.net/, 19 February 2014, date last accessed). The genomic DNA sequence contigs of LA 1589 were assigned to Heinz 1706 genome using local MUGSY^37^ downloaded from Sourceforge (http://mugsy.sourceforge.net/, 19 February 2014, date last accessed). InDel polymorphisms referring to Heinz 1706 were mined from the alignments using custom PERL scripts. Flanking sequences of 100 bp from each side of candidate InDels were extracted from Heinz 1706 sequences for insertion and LA 1589 sequences for deletion. The flanking sequences were then blasted against LA 1589 sequences for deletion or Heinz 1706 sequences for insertion using local BLASTall with an *E*-value of *e*^−20^ to remove hits with low similarity. The types (insertion or deletion), lengths, nucleotides, and chromosomal positions of InDels were extracted using a PERL script with the highest score of blast search.

### 2.3. Selection of InDels for validation and primer design

Our initial goal was to verify 3000 candidate InDels of 2 bp or longer evenly distributing on 12 chromosomes. Based on the genome sequenced for Heinz 1706 (760 Mb),^34^ the average distance between two adjacent InDels would be ∼250 kb. The number of InDels to be validated was determined by the length of each chromosome (Table 2). However, we found that the InDels were not always evenly distributed on chromosomes and hotspots have high levels of InDels than other regions. Therefore, we tried to acquire an InDel per 200 kb in each chromosome using a PERL script. If a region on a chromosome did not have InDel variation, the PERL script would make 200 plus 100 kb on circulation until it matched.

**Table 2.** Summary statistics for primer design, PCR amplification, and polymorphisms

Chromosome	Sequence length (∼Mb)^a^	No. of primers designed	No. of primers without PCR amplification	No. of primers without polymorphism	No. of primers examined	No. (percentage) of polymorphic InDels		
						*S. pimpinellifolium*	*S. lycopersicum* var. *cerasiforme*	*S. lycopersicum*
chr01	90.3	362	63	89	210	132 (62.9)	38 (18.1)	22 (10.5)
chr02	49.9	207	10	22	175	98 (56.0)	134 (76.6)	75 (42.9)
chr03	64.8	254	19	31	204	123 (60.3)	128 (62.7)	32 (15.7)
chr04	64.1	254	12	24	218	120 (55.0)	128 (58.7)	144 (66.1)
chr05	65.0	262	33	53	176	112 (63.6)	125 (71.0)	127 (72.2)
chr06	46.0	181	20	40	121	99 (81.8)	83 (68.6)	73 (60.3)
chr07	65.3	259	39	15	205	94 (45.9)	26 (12.7)	17 (8.3)
chr08	63.0	252	23	21	208	160 (76.9)	12 (5.8)	11 (5.3)
chr09	67.7	267	21	34	212	135 (63.7)	80 (37.7)	66 (31.1)
chr10	64.8	255	14	35	206	131 (63.6)	111 (53.9)	11 (5.3)
chr11	53.4	214	8	35	171	80 (46.8)	123 (71.9)	109 (63.7)
chr12	65.5	262	10	86	166	116 (69.9)	40 (24.1)	30 (18.1)
Total	759.8	3029	272	485	2272	1400 (61.6)	1028 (45.2)	717 (31.6)

^a^The sequenced genome size was obtained from Sato *et al.*^34^

To design primers for PCR validation of InDels, flanking sequences of 100 bp for each side of candidate InDels were extracted. Primers were designed using local Primer3^38^ downloaded from Sourceforge (http://sourceforge.net/project/showfiles.php?group_id=112461, 19 February 2014, date last accessed) with PCR product length 100–200 bp and the optimal length of primer sequence of 20 bp. Several primer pairs were designed for each InDel. The best primer pair was selected based on the optimal GC content of 40–60% and the difference of GC content between forward and reverse primers <10%. All the process was carried out using custom PERL scripts. Primers were synthesized at Sunbiotech Company (Beijing, China) or Sangong Company (Beijing, China).

### 2.4. Validation of InDels using PCR

The PCR technique was adapted to validate the candidate InDels. All synthesized primers were first used to amplify genomic DNA of tomato lines LA 1589 and Heinz 1706. Only primers that successfully amplified a product and had length polymorphisms were then used to detect polymorphisms in the 22 tomato genotypes.

All PCRs were done in 10-μl reaction volume using the method described in Wei *et al.*^39^ Reactions were heated at 95°C for 5 min, followed by 32 cycles of 30 s at 95°C, 30 s at 50–60°C depending on the *T*_m_ values of primer pairs, and 30 s at 72°C, with a final extension of 5 min at 72°C. The PCR products were subsequently separated in 8% polyacrylamide gel and visualized using the silver-staining approach.^17^

### 2.5. Data collection and analysis

The presence or absence of each allele for each InDel was coded by 1 or 0, respectively, and scored for a binary data matrix. Allele frequency of each InDel marker was calculated for each genotype. Nei's genetic distance^40^ was calculated for each pair of tomato genotypes using the programme in the software package PHYLIP 3.695 (http://evolution.genetics.washington.edu/phylip.html, 19 February 2014, date last accessed). An Unweighted Pair Group Method with Arithmetic Mean (UPGMA) cluster analysis was performed to develop a dendrogram.

The occurrences of InDels in coding regions of genes were examined by blasting the flanking sequences of 100 bp for each side of the InDel against the tomato ITAG2.3_cds.fasta downloaded from SGN using a PERL script.

## 3. Result

### 3.1. Candidate InDels between LA 1589 and Heinz 1706

A total of 145 695 candidate InDels were identified between the genome sequences of Heinz 1706 and LA 1589, of which 65 619 were insertions and 80 076 were deletions in Heinz 1706 (Table 3). The average size of predicted InDels was 4.1 bp with a range of 1–94 bp, of which ∼54.0% were 1 bp, 42.3% were 2–20 bp, and 3.7% were longer than 20 bp. The average density of InDels was one per 5.22 kb with a range of 4.33–6.72 kb on 12 chromosomes. The highest density was on chromosome 6 and the lowest density was on chromosome 12 (Table 3). The least difference of numbers for InDels between 1 bp and >1 bp was observed on chromosome 2 (101), while the largest was on chromosome 10 (1496).

**Table 3.** Predicted number and frequency of InDels between Heinz 1706 and LA 1589

Chromosome	No. of predicted InDels			Frequency of InDels (kb/InDel)		
	Total	1 bp	>1 bp	Total	1 bp	>1 bp
chr01	16 547	8777	7770	5.46	10.29	11.62
chr02	10 695	5398	5297	4.67	9.24	9.42
chr03	12 842	6779	6063	5.05	9.56	10.69
chr04	11 495	6112	5383	5.58	10.49	11.91
chr05	12 148	6816	5332	5.35	9.54	12.19
chr06	10 619	5540	5079	4.33	8.30	9.06
chr07	13 426	7386	6040	4.86	8.84	10.81
chr08	13 776	7591	6185	4.57	8.30	10.19
chr09	11 417	6251	5166	5.93	10.83	13.10
chr10	13 390	7443	5947	4.84	8.71	10.90
chr11	9587	5221	4366	5.57	10.23	12.23
chr12	9753	5432	4321	6.72	12.06	15.16
Total	145 695	78 746	66 949			
Average				5.22	9.65	11.35

### 3.2. Number of primers designed and success of PCR amplification

Using the approach described in the section ‘Selection of InDels for validation and primer design’ of Materials and methods, 3029 candidate InDels were selected and primers were designed for PCR validation (Supplementary Table S1). The average physical distance between two adjacent InDels was 250 kb with a range of 241 (chromosome 2) to 255 kb (chromosome 3) on 12 chromosomes. PCR results showed that 272 primer pairs could not generate PCR products from the genomic DNA of both Heinz 1706 and LA 1589 (Table 2) . The PCR success rate was 91.0%, which was consistent with our previous finding of 91.9% for PCR amplification of genomic DNA in tomato.^9^ The InDel sizes of PCR products amplified by most primer pairs (98.5%) were as predicted. However, 23 primer pairs showed smaller and 10 primer pairs showed larger sizes than predicted (Supplementary Table S1). In addition, 485 primer pairs did not show detectable polymorphisms between Heinz 1706 and LA 1589 (Table 2). The InDel sizes between 6 and 30 bp had a high percentage (83.6%) of polymorphism validation, while InDels with sizes of <6 bp and >30 bp received 78.3 or 43.3% polymorphism validation, respectively. Particularly, only one of five InDels was validated when the size was >50 bp (Supplementary Table S2). The primer pairs with PCR failure or non-detectable polymorphisms were excluded, and the remaining 2272 primer pairs were used for subsequent analysis. Therefore, the actual average distance between two adjacent InDels was 334 kb with a range of 285 (chromosome 2) to 430 kb (chromosome 1) on 12 chromosomes.

**Figure 1.**
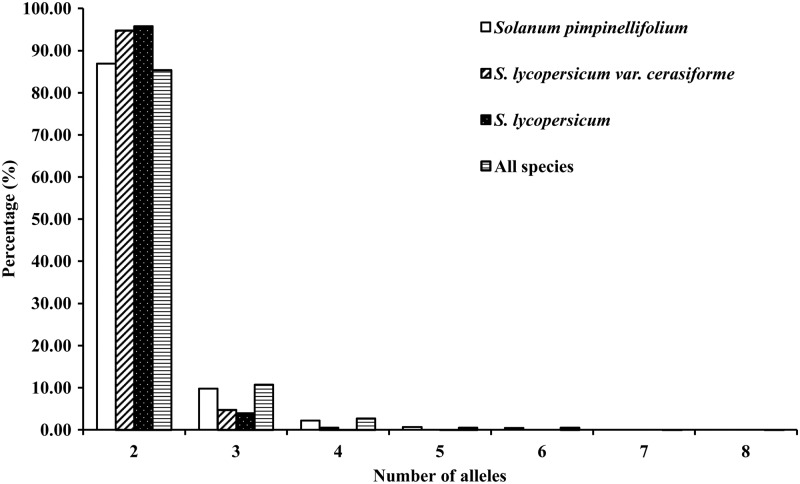
Frequency distribution of InDels (≥2 bp) in *Solanum pimpinellifolium*, *S. lycopersicum* var. *cerasiforme*, and *S. lycopersicum*.

The 2272 InDel markers generated 5025 alleles in the whole collection of 22 tomato genotypes. The number of alleles generated for all InDels varied from 2 to 8 with an average of 2.2. Among the polymorphic InDels, most (85.3%) had two alleles, 10.7% had three alleles, and 2.7% had four alleles (Fig. 1). Only three and two markers had seven and eight alleles, respectively. Similarly, 84.9% polymorphic InDels in *S. pimpinellifolium*, 94.7% in *S. lycopersicum* var. *cerasiforme*, and 95.8% in *S. lycopersicum* had two alleles (Fig. 1).

### 3.3. Marker polymorphisms and distribution among three tomato species

**Figure 2.**
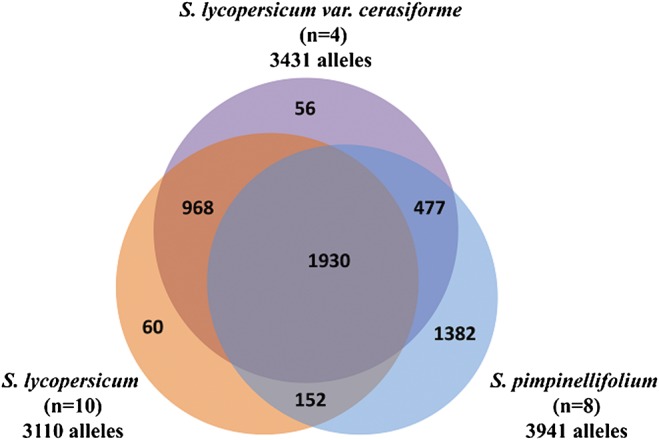
Venn diagram shows the proportion of common alleles among *Solanum pimpinellifolium*, *S. lycopersicum* var. *cerasiforme*, and *S. lycopersicum***.** This figure appears in colour in the online version of *DNA Research*.

Of the 5025 alleles amplified by 2272 InDel markers, 1930 were shared by all three species. The total number of alleles in each species reduced from 3941 in *S. pimpinellifolium* to 3431 in *S. lycopersicum* var. *cerasiforme* and 3110 in *S. lycopersicum* (Fig. 2). The number of alleles unique to each species also dramatically decreased from 1382 in *S. pimpinellifolium* to 56 in *S. lycopersicum* var. *cerasiforme* and 60 in *S. lycopersicum. Solanum pimpinellifolium* shared more alleles with *S. lycopersicum* var. *cerasiforme* than with *S. lycopersicum*.

**Figure 3.**
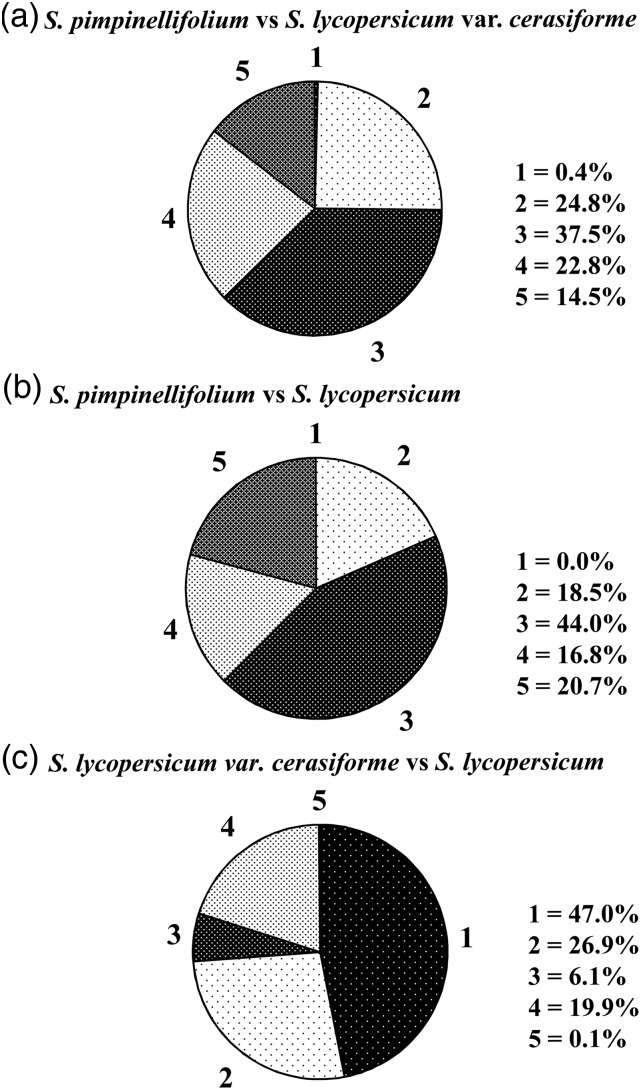
Pairwise comparisons of allelic variation among *Solanum pimpinellifolium*, *S. lycopersicum* var. *cerasiforme*, and *S. lycopersicum*. Pie diagrams show the proportion of 2272 InDels that fell into five categories: (1) InDels where a monomorphic allele was shared by all members in the two species; (2) InDels where alleles were found among the members of the two species; (3) InDels where a unique allele was found among members of the first species listed, whereas an alternative allele (found in both groups) was fixed in the second species; (4) InDels where a unique allele was found among members of the second species listed, whereas an alternative allele (found in both species) was fixed in the first species; (5) InDels where the two species were fixed for alternative alleles.

Pairwise comparisons revealed that almost all InDel markers were polymorphic between *S. pimpinellifolium* and *S. lycopersicum* var. *cerasiforme* or *S. lycopersicum*. However, the proportion of polymorphic InDels reduced to 53.0% between *S. lycopersicum* var. *cerasiforme* and *S. lycopersicum*. There were 0.1–20.7% InDels had alleles alternatively fixed in paired species. In addition, 18.5–26.9% InDels had alleles shared by paired species. Proportions of InDels with alleles specific to one certain species varied from 6.1 to 44.0% (Fig. 3). The proportion of polymorphic InDels was 61.4–100.0% (average 84.6%) between any accession in *S. pimpinellifolium* and any genotype in *S. lycopersicum*, 55.3–93.8% (average 71.5%) between any accession in *S. pimpinellifolium* and any line in *S. lycopersicum* var. *cerasiforme*, and 7.7–33.9% (average 19.2%) between any line in *S. lycopersicum* var. *cerasiforme* and any genotype in *S. lycopersicum* (Supplementary Table S3).

Although the 2272 InDels almost evenly distributed across all 12 chromosomes (Supplementary Fig. S1), the distribution of polymorphic markers varied for three species (Supplementary Fig. S2). *Solanum pimpinellifolium* had a relatively even distribution of polymorphic InDels on all 12 chromosomes. *Solanum lycopersicum* var. *cerasiforme* had the similar distribution pattern of polymorphic InDels as *S. pimpinellifolium* on chromosomes 2, 3, 4, 5, 6, 9, 10, and 11, but clusters of polymorphic InDels occurred at some regions on chromosomes 1, 7, and 12. The distribution of polymorphic InDels varied across and within chromosomes in *S. lycopersicum*. Among six chromosomes with less polymorphic InDels, chromosomes 1, 8, 10, and 12 had relatively even distribution, while the long-arm ends of chromosomes 3 and 7 had more InDels than other regions. There were less InDels at one end of chromosomes 2, 4, 5, 9, and 11. However, chromosomes 5, 9, and 11 showed relatively even distribution. On chromosome 6, the short arm had more polymorphic InDels than the long arm.

**Figure 4.**
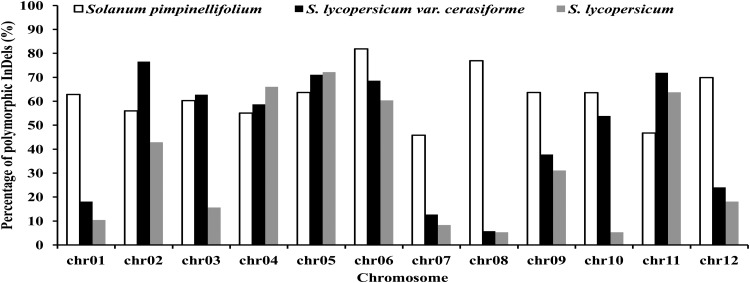
Distribution of the proportion of polymorphic InDels on 12 chromosomes in *Solanum pimpinellifolium*, *S. lycopersicum* var. *cerasiforme*, and *S. lycopersicum*.

The proportion of polymorphic InDels on 12 chromosomes ranged from 45.9 to 81.8% in *S. pimpinellifolium*, 5.8 to 76.6% in *S. lycopersicum* var. *cerasiforme*, and 5.3 to 72.2% in *S. lycopersicum* (Fig. 4). The numbers of polymorphic InDels considerably decreased on four chromosomes 1, 7, 8, and 12 in *S. lycopersicum* var. *cerasiforme* and *S. lycopersicum* (Table 2). Furthermore, the proportions of polymorphic InDels on chromosomes 3 and 10 were close between *S. pimpinellifolium* and *S. lycopersicum* var. *cerasiforme*, but significantly decreased in *S. lycopersicum* (Fig. 4). Interestingly, increases of InDel polymorphisms were observed on chromosomes 4, 5, and 11 in *S. lycopersicum* var. *cerasiforme* and *S. lycopersicum*. The proportions of polymorphic InDels also increased on chromosomes 2 and 3 in *S. lycopersicum* var. *cerasiforme*.

### 3.4. Marker polymorphisms and genetic vitiation within three tomato species

The proportion of polymorphic InDels was 61.6% in 8 *S. pimpinellifolium* accessions, 45.2% in 4 *S. lycopersicum* var. *cerasiforme* accessions, and 31.6% in 10 cultivated tomato varieties (Table 2). However, the rate of polymorphic InDels between any two genotypes was low with a range of 14.3–33.6% in *S. pimpinellifolium*, 17.5–31.5% in *S. lycopersicum* var. *cerasiforme*, and 1.5–19.8% in *S. lycopersicum* (Supplementary Table S3).

Not surprisingly, the eight accessions of *S. pimpinellifolium* had the largest genetic variation among three species. The average genetic distance was 0.216 with a range from 0.178 (PI 128216) to 0.244 (LA1589). Accessions LA 1589 and LA 2181 had the greatest genetic distance with 0.394, whereas accessions PI 128216 and LA 0373 had the least genetic distance with 0.137. The average genetic distance slightly reduced to 0.202 with a range from 0.162 (LA 4133) to 0.237 (PI 114490) in four *S. lycopersicum* var. *cerasiforme* lines, but significantly decreased to 0.108 with a range of 0.086 (Baiguoqiangfeng) to 0.139 (M 82) in 10 varieties of *S. lycopersicum*. The minimum genetic distance was 0.012 between varieties Liger 87-5 and M 82, followed by 0.015 between varieties Baiguoqiangfeng and Zhongshu 5, while the largest genetic distance was 0.214 between Shijifeng and M 82.

**Figure 5.**
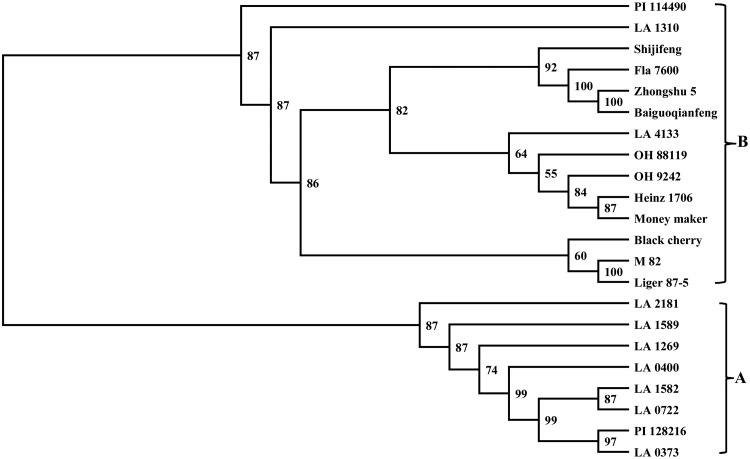
The dendrogram of 22 tomato genotypes based on 2272 InDel marker data, and generated from Nei's genetic distance matrix by UPGMA in PHYLIP 3.695.

The dendrogram was constructed from the pairwise genetic distance matrices based on Nei's distance for 22 genotypes. Two distinct groups, A and B, were obtained (Fig. 5). All 8 accessions of *S. pimpinellifolium* were in Group A, and 10 *S. lycopersicum* var. *cerasiforme* cultivars and 4 *S. lycopersicum* var. *cerasiforme* accessions were in Group B. The four fresh market cultivars clustered together. However, five processing varieties, one greenhouse variety, and four *S. lycopersicum* var. *cerasiforme* accessions did not form their own clades. Of the four *S. lycopersicum* var. *cerasiforme* lines, LA 4133 clustered to three processing and one greenhouse varieties, Black cherry clustered to two processing varieties, while PI 114490 and LA 1310 stood alone.

### 3.5. Genes with InDels in the coding region

Blast search of flanking sequences of 2272 validated InDels against the tomato ITAG2.3_cds.fasta data identified 56 InDels in coding regions of annotated genes (Supplementary Table S4), of which 64.3% were deletions in Heinz 1706 and 35.7% were insertion in Heinz 1706. Based on the sizes of InDels, 28.6% of InDels were frame-shift mutations, because the numbers of nucleotides in the InDels were indivisible by three. The remaining 71.4% InDels did not result in frame-shift, but would cause insertion or deletion of some amino acids.

## 4. Discussion

Molecular markers are important to genetic study and marker-assisted selection. Large-scale discovery combining high-throughput genotyping of SNPs have shown its power in gene identification and breeding in tomato.^12^ However, high costs and technical or equipment demands will still be a major obstacle for large-scale use of SNPs in the developing countries.^41,42^ On the contrary, the genotyping of short InDels is relatively easy and inexpensive with a simple PCR and electrophoresis. Short InDels can also be analysed with high-throughput technologies^26,43,44^ and in large-scale multiplexing.^45^ As a type of genetic markers, InDels have been successfully used for forensic analysis^46–48^ and individual identification^44,45^ in human, as well as genetic studies in several plant species including rice, wheat, citrus, and Arabidopsis.^33^ Although the tomato genome sequences have been widely used in various purposes including SNP discovery, genetic mapping, gene prediction, gene expression, genetic diversity, comparative genomics, and epigenetics since their release,^49^ identification of InDels has so far been confined to detect polymorphisms between wild species and cultivated tomato.^34,35^ In this study, we identified InDels by comparative analysis of genome sequences between *S. pimpinellifolium* and *S. lycopersicum*, and then validated them in 10 cultivated tomato lines via PCR amplification. Of 2272 InDels polymorphic between LA 1589 and Heinz 1706, 31.6% were polymorphic among the 10 cultivated tomato varieties and 1.5–19.8% were polymorphic between any 2 of the 10 cultivated tomato varieties. Based on the total number of InDels (145 695) between LA 1589 and Heinz 1706, we estimated that there were 2100–28 800 InDels between any two cultivated tomato varieties, suggesting that there were abundant InDels for genetic study and marker-assisted selection in the cultivated tomato.

Precise identification of InDels in sequence databases depends on the strategy and the parameters used for data mining as well as the quality of sequence data. Since InDels are the dominant error type generated by 454 pyrosequencing^50^ and an InDel error rate of one per 6.4 kb was observed in tomato,^34^ the initial work on identification of InDels between the genomes of LA 1589 and Heinz 1706 did not count InDels of 1 and 2 bp to avoid overestimation of small InDels due to sequencing errors.^34^ Using a bioinformatic pipeline involving various comparative genomics tools, 9474 InDels of 15–100 bp were identified between LA 1589 and Heinz 1706, and >80% could be verified by PCR (Jiang *et al.* unpublished data, acquired from ftp://ftp.solgenomics.net/maps_and_markers/LippmanZ/, 19 February 2014, date last accessed). In this study, a total of 145 695 InDels were predicted between LA 1589 and Heinz 1796, which was approximate one-fifth of 749 966 InDels Identified in Sato *et al*.^34^ The overall frequency of InDels (one per 5.22 kb) was also much lower than one per 110 bp in Sato *et al*.^34^ However, the number (9137) of InDels of 15–94 bp was close to the results of Jiang *et al.*, though the strategies used for InDels identification were different. Owing to the lack of methodology description in Sato *et al.*,^34^ we were not able to determine the cause of the difference between two studies. Two points might be worthy of notice. First, the lengths of putative InDels identified in two studies were different with ranges of 3–300 bp in Sato *et al.*^34^ and 1–94 bp in this study. We could not identify any InDels >94 bp using our methodology. Secondly, the rate of validation (82.4%) was close to 81.7% obtained in Koenig *et al.*,^35^ though the comparisons involved in different wild species and cultivated varieties, indicating that ∼20% of predicted InDels (≥2 bp) were false due to sequencing error. All these suggested that our prediction might be more close to the real number of InDels in the currently available genome sequences of LA 1589 and Heinz 1706.

The polymorphic InDels evenly distributed across all 12 chromosomes in *S. pimpinellifolium*, but appeared non-randomly distributed across and within chromosomes in *S. lycopersicum* var. *cerasiforme* and *S. lycopersicum*. Domestication and selection could be one causal of this difference. For example, there were 38 and 35 polymorphic InDels at the bottom (∼11 Mb) of chromosome 2 in *S. pimpinellifolium* and *S. lycopersicum* var. *cerasiforme*, respectively, but only two InDels were polymorphic in *S. lycopersicum*. This might be due to the existence of quantitative trait loci for fruit weight and selection for large fruit in *S. lycopersicum*.^12^ In addition, several studies have proved that the introgression of disease resistance genes in many cultivars has strong influence on SNP patterns.^19,51^ This kind of introgression could also cause the difference of polymorphic InDels distribution among three species.

It has been suggested that domestication and inbreeding dramatically reduced the genetic variation^52^ and modern cultivars have less genetic variation than old ones in tomato.^53,54^ In this study, genetic variation of three species was investigated using the same large set of InDel markers, which allowed us to compare genetic polymorphisms among and within species at the same time. The number of polymorphic InDels, the total number of alleles amplified by InDel markers, and the average genetic distance in 10 *S. lycopersicum* varieties significantly reduced comparing with those in 8 *S. pimpinellifolium* accessions, supported the reduction of genetic variation in cultivated tomato. The four *S. lycopersicum* var. *cerasiforme* accessions showed an intermediate amount of genetic diversity between *S. lycopersicum* and *S. pimpinellifolium*, which was consistent with previous findings.^55,56^ However, some novel alleles occurred in both *S. lycopersicum* var. *cerasiforme* and *S. lycopersicum*, suggesting that domestication and selection could also generate new variation.

The occurrence of InDels in coding regions of a gene can either cause frame-shift or amino acid InDels, which most likely alternates the gene function and results in phenotype change.^57^ A *Rider* mutational insertion event occurring in the first exon of the *Psy1* gene causes the early termination of *Psy1* transcription that results in yellow flesh in the tomato *r* mutant.^58^ A single-base deletion mutation in the coding region of *SlIAA9* gene, an *Aux/IAA* gene involving in tomato leaf morphology, converts tomato compound leaves to simple leaves.^59^ InDels occurring in the promoter region can also affect the gene expression.^60^ Here, we identified 145 695 InDels between LA 1589 and Heinz 1706, and 31.6% of them were polymorphic in cultivated tomatoes. The percentage of InDels (2.5%) occurring in coding regions of genes identified in this study was much lower than our recent work (19.7%) on comparative analysis of resistance-like genes between LA 1589 and Heinz 1706.^61^ Identification of specific genes in our previous work other than a random sample in this study could cause the different proportions of InDels in coding regions.

In conclusion, there are abundant short InDels in cultivated tomato. Identification and validation of this kind of short InDels will not only provide molecular markers for genetic study and marker-assisted selection in breeding, but also provide useful information for gene cloning and functional analysis.

## Supplementary data

Supplementary data are available at www.dnaresearch.oxfordjournals.org.

## Funding

The work was partially supported by the National Program on Key Basic Research Projects (The 973 Program: 2012CB113900) and the National Natural Science Foundation of China (31171973).

## Supplementary Material

Supplementary Data
