# Supplementary material for: In Silico Identification and Experimental Validation of Insertion–Deletion Polymorphisms in Tomato Genome
Source: DNA Res. 2014 Mar 11;21(4):429–38. doi: 10.1093/dnares/dsu008 (PMC4131836; doi:10.1093/dnares/dsu008)
Supplement: Supplementary Data [file supp_21_4_429__index.html]

In Silico Identification and Experimental Validation of Insertion–Deletion Polymorphisms in Tomato Genome — Supplementary Data 

# *In Silico* Identification and Experimental Validation of Insertion–Deletion Polymorphisms in Tomato Genome

## Supplementary Data

Supplementary Data

**Files in this Data Supplement:**

- Supplementary Figure 2 - pdf file
- Supplementary Data - Doc file
- Supplementary Figure 1 - tif file
- Supplementary Table 1 - xls file
- Supplementary Table 2 - xls file
- Supplementary Table 3 - xls file
- Supplementary Table 4 - xls file
