# Supplementary material for: In Silico Identification and Experimental Validation of Insertion–Deletion Polymorphisms in Tomato Genome
Source: DNA Res. 2014 Mar 11;21(4):429–38. doi: 10.1093/dnares/dsu008 (PMC4131836; doi:10.1093/dnares/dsu008)
Supplement: Supplementary Data [file supp_dsu008_dsu008supp_fig2.pdf]

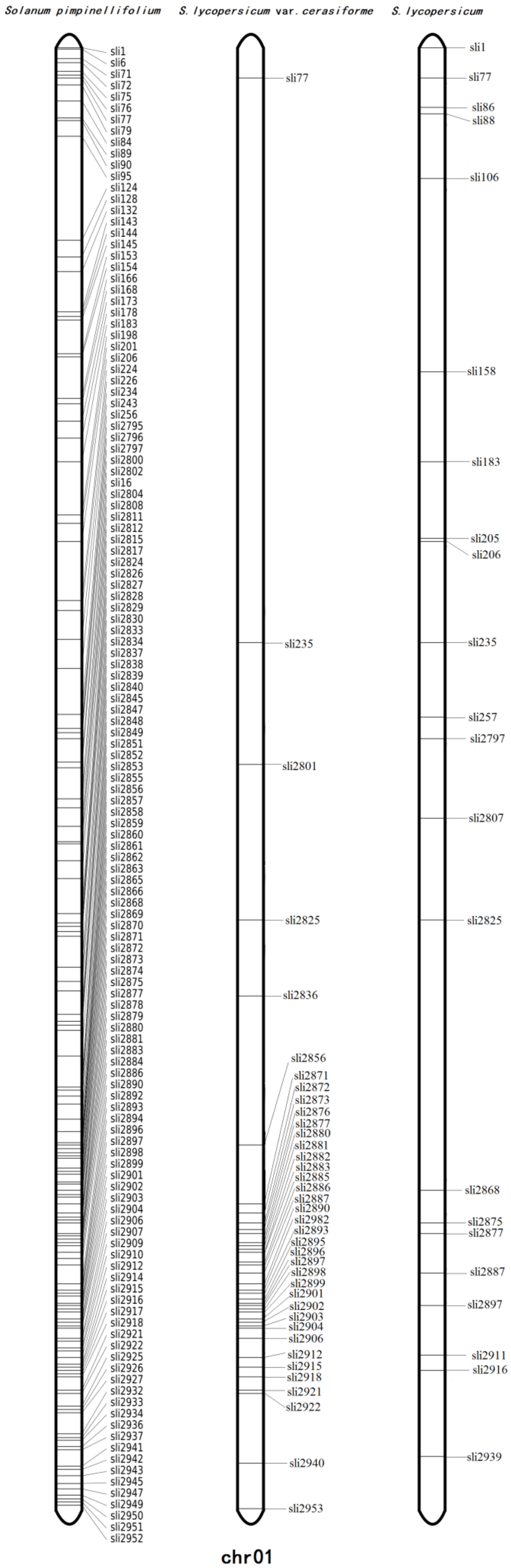
Solanum pimpinellifolium
sli1
sli6
sli71
sli72
sli75
sli76
sli77
sli79
sli84
sli89
sli90
sli95
sli124
sli128
sli132
sli143
sli144
sli145
sli153
sli154
sli166
sli168
sli173
sli178
sli183
sli198
sli201
sli206
sli224
sli226
sli234
sli243
sli256
sli2795
sli2796
sli2797
sli2800
sli2802
sli16
sli2804
sli2808
sli2811
sli2812
sli2815
sli2817
sli2824
sli2826
sli2827
sli2828
sli2829
sli2830
sli2833
sli2834
sli2837
sli2838
sli2839
sli2840
sli2845
sli2847
sli2848
sli2849
sli2851
sli2852
sli2853
sli2855
sli2856
sli2857
sli2858
sli2859
sli2860
sli2861
sli2862
sli2863
sli2865
sli2866
sli2868
sli2869
sli2870
sli2871
sli2872
sli2873
sli2874
sli2875
sli2877
sli2878
sli2879
sli2880
sli2881
sli2883
sli2884
sli2886
sli2890
sli2892
sli2893
sli2894
sli2896
sli2897
sli2898
sli2899
sli2901
sli2902
sli2903
sli2904
sli2906
sli2907
sli2909
sli2910
sli2912
sli2914
sli2915
sli2916
sli2917
sli2918
sli2921
sli2922
sli2925
sli2926
sli2927
sli2932
sli2933
sli2934
sli2936
sli2937
sli2941
sli2942
sli2943
sli2945
sli2947
sli2949
sli2950
sli2951
sli2952
S. lycopersicum var. cerasiforme
sli77
sli235
sli2801
sli2825
sli2836
sli2856
sli2871
sli2872
sli2873
sli2876
sli2877
sli2880
sli2881
sli2882
sli2883
sli2885
sli2886
sli2887
sli2890
sli2982
sli2893
sli2895
sli2896
sli2897
sli2898
sli2899
sli2901
sli2902
sli2903
sli2904
sli2906
sli2912
sli2915
sli2918
sli2921
sli2922
sli2940
sli2953
S. lycopersicum
sli1
sli77
sli86
sli88
sli106
sli158
sli183
sli205
sli206
sli235
sli257
sli2797
sli2807
sli2825
sli2868
sli2875
sli2877
sli2887
sli2897
sli2911
sli2916
sli2939

chr01

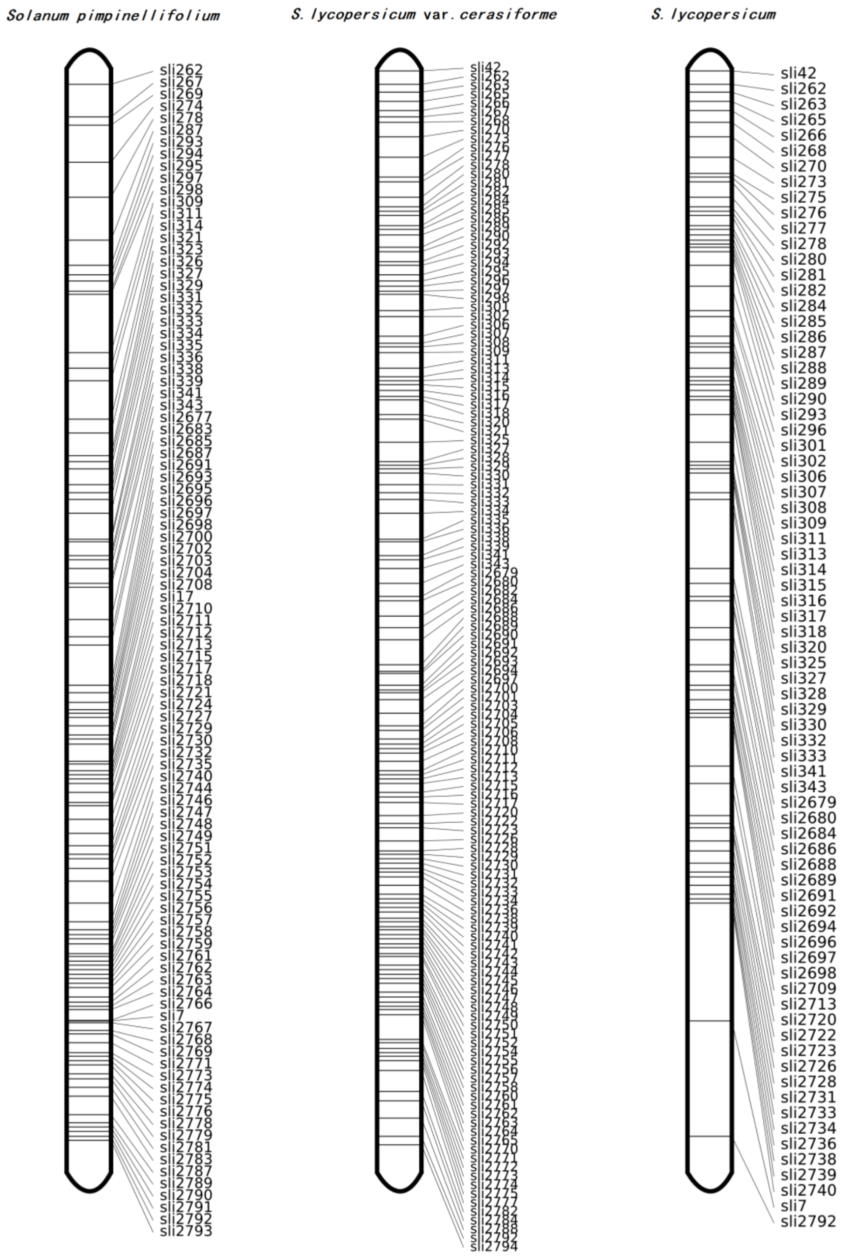
Solanum pimpinellifolium
S. lycopersicum var. cerasiforme
S. lycopersicum
sli262
sli267
sli269
sli274
sli278
sli287
sli293
sli294
sli295
sli297
sli298
sli309
sli311
sli314
sli321
sli323
sli326
sli327
sli329
sli331
sli332
sli333
sli334
sli335
sli336
sli338
sli339
sli341
sli343
sli2677
sli2683
sli2685
sli2687
sli2691
sli2693
sli2695
sli2696
sli2697
sli2698
sli2700
sli2702
sli2703
sli2704
sli2708
sli17
sli2710
sli2711
sli2712
sli2713
sli2715
sli2717
sli2718
sli2721
sli2724
sli2727
sli2729
sli2730
sli2732
sli2735
sli2740
sli2744
sli2746
sli2747
sli2748
sli2749
sli2751
sli2752
sli2753
sli2754
sli2755
sli2756
sli2757
sli2758
sli2759
sli2761
sli2762
sli2763
sli2764
sli2766
sli7
sli2767
sli2768
sli2769
sli2771
sli2773
sli2774
sli2775
sli2776
sli2778
sli2779
sli2781
sli2783
sli2787
sli2789
sli2790
sli2791
sli2792
sli2793
sli42
sli262
sli263
sli265
sli266
sli268
sli270
sli273
sli275
sli276
sli277
sli278
sli280
sli281
sli282
sli284
sli285
sli286
sli287
sli288
sli289
sli290
sli293
sli296
sli301
sli302
sli306
sli307
sli308
sli309
sli311
sli313
sli314
sli315
sli316
sli317
sli318
sli320
sli325
sli327
sli328
sli329
sli330
sli332
sli333
sli341
sli343
sli2679
sli2680
sli2684
sli2686
sli2688
sli2689
sli2691
sli2692
sli2694
sli2696
sli2697
sli2698
sli2709
sli2713
sli2720
sli2722
sli2723
sli2726
sli2728
sli2731
sli2733
sli2734
sli2736
sli2738
sli2739
sli2740
sli7
sli2792

chr02

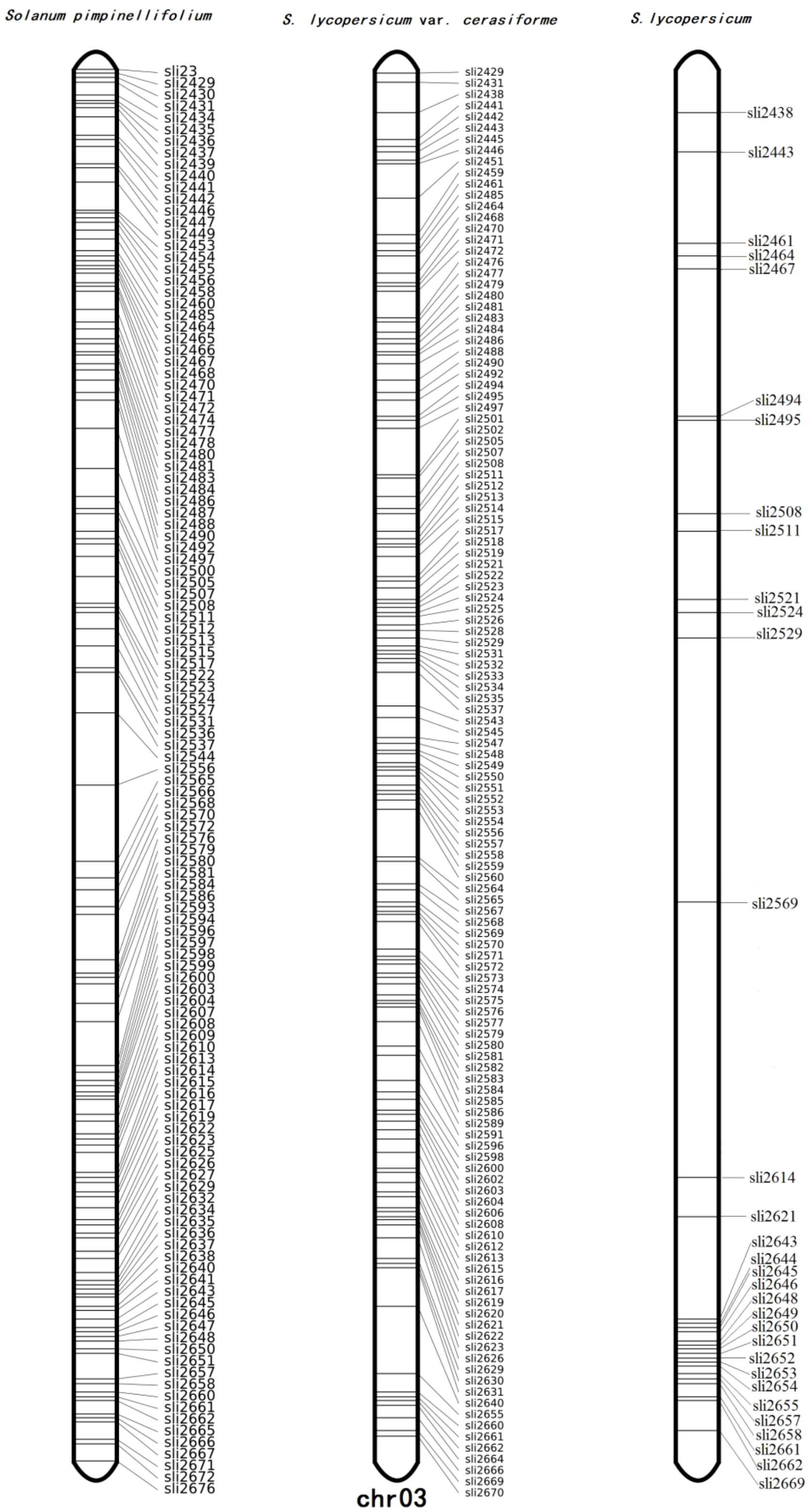

Solanum pimpinellifolium
S. lycopersicum var. cerasiforme
S. lycopersicum
sli23
sli2429
sli2430
sli2431
sli2434
sli2435
sli2436
sli2437
sli2439
sli2440
sli2441
sli2442
sli2446
sli2447
sli2449
sli2453
sli2454
sli2455
sli2456
sli2458
sli2460
sli2485
sli2464
sli2465
sli2466
sli2467
sli2468
sli2470
sli2471
sli2472
sli2474
sli2477
sli2478
sli2480
sli2481
sli2483
sli2484
sli2486
sli2487
sli2488
sli2490
sli2492
sli2497
sli2500
sli2505
sli2507
sli2508
sli2511
sli2512
sli2513
sli2515
sli2517
sli2522
sli2523
sli2524
sli2527
sli2531
sli2536
sli2537
sli2544
sli2556
sli2565
sli2566
sli2568
sli2570
sli2572
sli2576
sli2579
sli2580
sli2581
sli2584
sli2586
sli2593
sli2594
sli2596
sli2597
sli2598
sli2599
sli2600
sli2603
sli2604
sli2607
sli2608
sli2609
sli2610
sli2613
sli2614
sli2615
sli2616
sli2617
sli2619
sli2622
sli2623
sli2625
sli2626
sli2627
sli2629
sli2632
sli2634
sli2635
sli2636
sli2637
sli2638
sli2640
sli2641
sli2643
sli2645
sli2646
sli2647
sli2648
sli2650
sli2651
sli2657
sli2658
sli2660
sli2661
sli2662
sli2665
sli2666
sli2667
sli2671
sli2672
sli2676
sli2429
sli2431
sli2438
sli2441
sli2442
sli2443
sli2445
sli2446
sli2451
sli2459
sli2461
sli2485
sli2464
sli2468
sli2470
sli2471
sli2472
sli2476
sli2477
sli2479
sli2480
sli2481
sli2483
sli2484
sli2486
sli2488
sli2490
sli2492
sli2494
sli2495
sli2497
sli2501
sli2502
sli2505
sli2507
sli2508
sli2511
sli2512
sli2513
sli2514
sli2515
sli2517
sli2518
sli2519
sli2521
sli2522
sli2523
sli2524
sli2525
sli2526
sli2528
sli2529
sli2531
sli2532
sli2533
sli2534
sli2535
sli2537
sli2543
sli2545
sli2547
sli2548
sli2549
sli2550
sli2551
sli2552
sli2553
sli2554
sli2556
sli2557
sli2558
sli2559
sli2560
sli2564
sli2565
sli2567
sli2568
sli2569
sli2570
sli2571
sli2572
sli2573
sli2574
sli2575
sli2576
sli2577
sli2579
sli2580
sli2581
sli2582
sli2583
sli2584
sli2585
sli2586
sli2589
sli2591
sli2596
sli2598
sli2600
sli2602
sli2603
sli2604
sli2606
sli2608
sli2610
sli2612
sli2613
sli2615
sli2616
sli2617
sli2619
sli2620
sli2621
sli2622
sli2623
sli2626
sli2629
sli2630
sli2631
sli2640
sli2655
sli2660
sli2661
sli2662
sli2664
sli2666
sli2669
sli2670
chr03
sli2438
sli2443
sli2461
sli2464
sli2467
sli2494
sli2495
sli2508
sli2511
sli2521
sli2524
sli2529
sli2569
sli2614
sli2621
sli2643
sli2644
sli2645
sli2646
sli2648
sli2649
sli2650
sli2651
sli2652
sli2653
sli2654
sli2655
sli2657
sli2658
sli2661
sli2662
sli2669

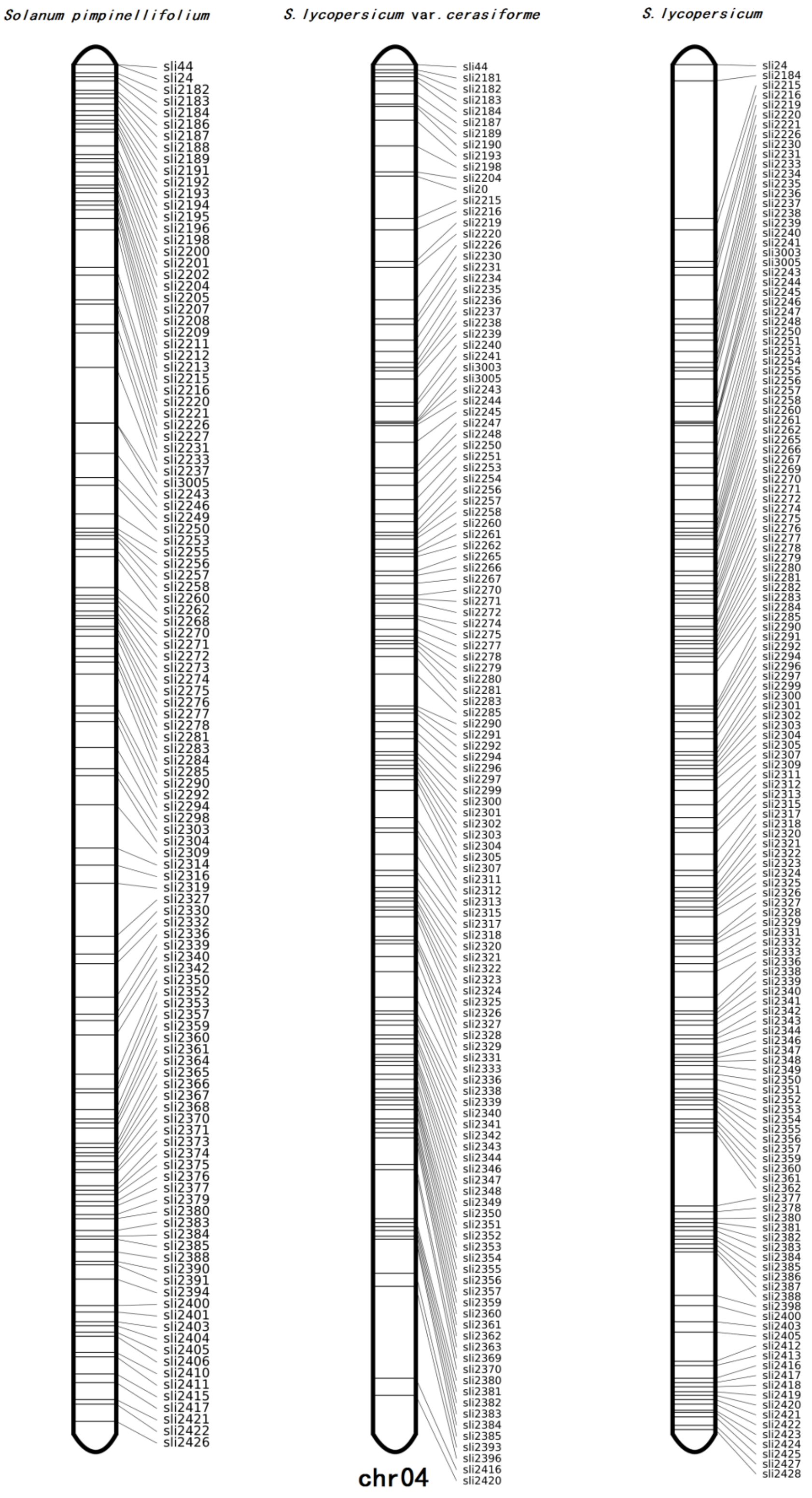

Solanum pimpinellifolium
S. lycopersicum var. *cerasiforme*
S. lycopersicum
sli44 sli24 sli2182 sli2183 sli2184 sli2186 sli2187 sli2188 sli2189 sli2191 sli2192 sli2193 sli2194 sli2195 sli2196 sli2198 sli2200 sli2201 sli2202 sli2204 sli2205 sli2207 sli2208 sli2209 sli2211 sli2212 sli2213 sli2215 sli2216 sli2220 sli2221 sli2226 sli2227 sli2231 sli2233 sli2237 sli3005 sli2243 sli2246 sli2249 sli2250 sli2253 sli2255 sli2256 sli2257 sli2258 sli2260 sli2262 sli2268 sli2270 sli2271 sli2272 sli2273 sli2274 sli2275 sli2276 sli2277 sli2278 sli2281 sli2283 sli2284 sli2285 sli2290 sli2292 sli2294 sli2298 sli2303 sli2304 sli2309 sli2314 sli2316 sli2319 sli2327 sli2330 sli2332 sli2336 sli2339 sli2340 sli2342 sli2350 sli2352 sli2353 sli2357 sli2359 sli2360 sli2361 sli2364 sli2365 sli2366 sli2367 sli2368 sli2370 sli2371 sli2373 sli2374 sli2375 sli2376 sli2377 sli2379 sli2380 sli2383 sli2384 sli2385 sli2388 sli2390 sli2391 sli2394 sli2400 sli2401 sli2403 sli2404 sli2405 sli2406 sli2410 sli2411 sli2415 sli2417 sli2421 sli2422 sli2426
sli44 sli2181 sli2182 sli2183 sli2184 sli2187 sli2189 sli2190 sli2193 sli2198 sli2204 sli20 sli2215 sli2216 sli2219 sli2220 sli2226 sli2230 sli2231 sli2234 sli2235 sli2236 sli2237 sli2238 sli2239 sli2240 sli2241 sli3003 sli3005 sli2243 sli2244 sli2245 sli2247 sli2248 sli2250 sli2251 sli2253 sli2254 sli2256 sli2257 sli2258 sli2260 sli2261 sli2262 sli2265 sli2266 sli2267 sli2270 sli2271 sli2272 sli2274 sli2275 sli2277 sli2278 sli2279 sli2280 sli2281 sli2283 sli2285 sli2290 sli2291 sli2292 sli2294 sli2296 sli2297 sli2299 sli2300 sli2301 sli2302 sli2303 sli2304 sli2305 sli2307 sli2311 sli2312 sli2313 sli2315 sli2317 sli2318 sli2320 sli2321 sli2322 sli2323 sli2324 sli2325 sli2326 sli2327 sli2328 sli2329 sli2331 sli2333 sli2336 sli2338 sli2339 sli2340 sli2341 sli2342 sli2343 sli2344 sli2346 sli2347 sli2348 sli2349 sli2350 sli2351 sli2352 sli2353 sli2354 sli2355 sli2356 sli2357 sli2359 sli2360 sli2361 sli2362 sli2363 sli2369 sli2370 sli2380 sli2381 sli2382 sli2383 sli2384 sli2385 sli2393 sli2396 sli2416 sli2420
chr04
sli24 sli2184 sli2215 sli2216 sli2219 sli2220 sli2221 sli2226 sli2230 sli2231 sli2233 sli2234 sli2235 sli2236 sli2237 sli2238 sli2239 sli2240 sli2241 sli3003 sli3005 sli2243 sli2244 sli2245 sli2246 sli2247 sli2248 sli2250 sli2251 sli2253 sli2254 sli2255 sli2256 sli2257 sli2258 sli2260 sli2261 sli2262 sli2265 sli2266 sli2267 sli2269 sli2270 sli2271 sli2272 sli2274 sli2275 sli2276 sli2277 sli2278 sli2279 sli2280 sli2281 sli2282 sli2283 sli2284 sli2285 sli2290 sli2291 sli2292 sli2294 sli2296 sli2297 sli2299 sli2300 sli2301 sli2302 sli2303 sli2304 sli2305 sli2307 sli2309 sli2311 sli2312 sli2313 sli2315 sli2317 sli2318 sli2320 sli2321 sli2322 sli2323 sli2324 sli2325 sli2326 sli2327 sli2328 sli2329 sli2331 sli2332 sli2333 sli2336 sli2338 sli2339 sli2340 sli2341 sli2342 sli2343 sli2344 sli2346 sli2347 sli2348 sli2349 sli2350 sli2351 sli2352 sli2353 sli2354 sli2355 sli2356 sli2357 sli2359 sli2360 sli2361 sli2362 sli2377 sli2378 sli2380 sli2381 sli2382 sli2383 sli2384 sli2385 sli2386 sli2387 sli2388 sli2398 sli2400 sli2403 sli2405 sli2412 sli2413 sli2416 sli2417 sli2418 sli2419 sli2420 sli2421 sli2422 sli2423 sli2424 sli2425 sli2427 sli2428

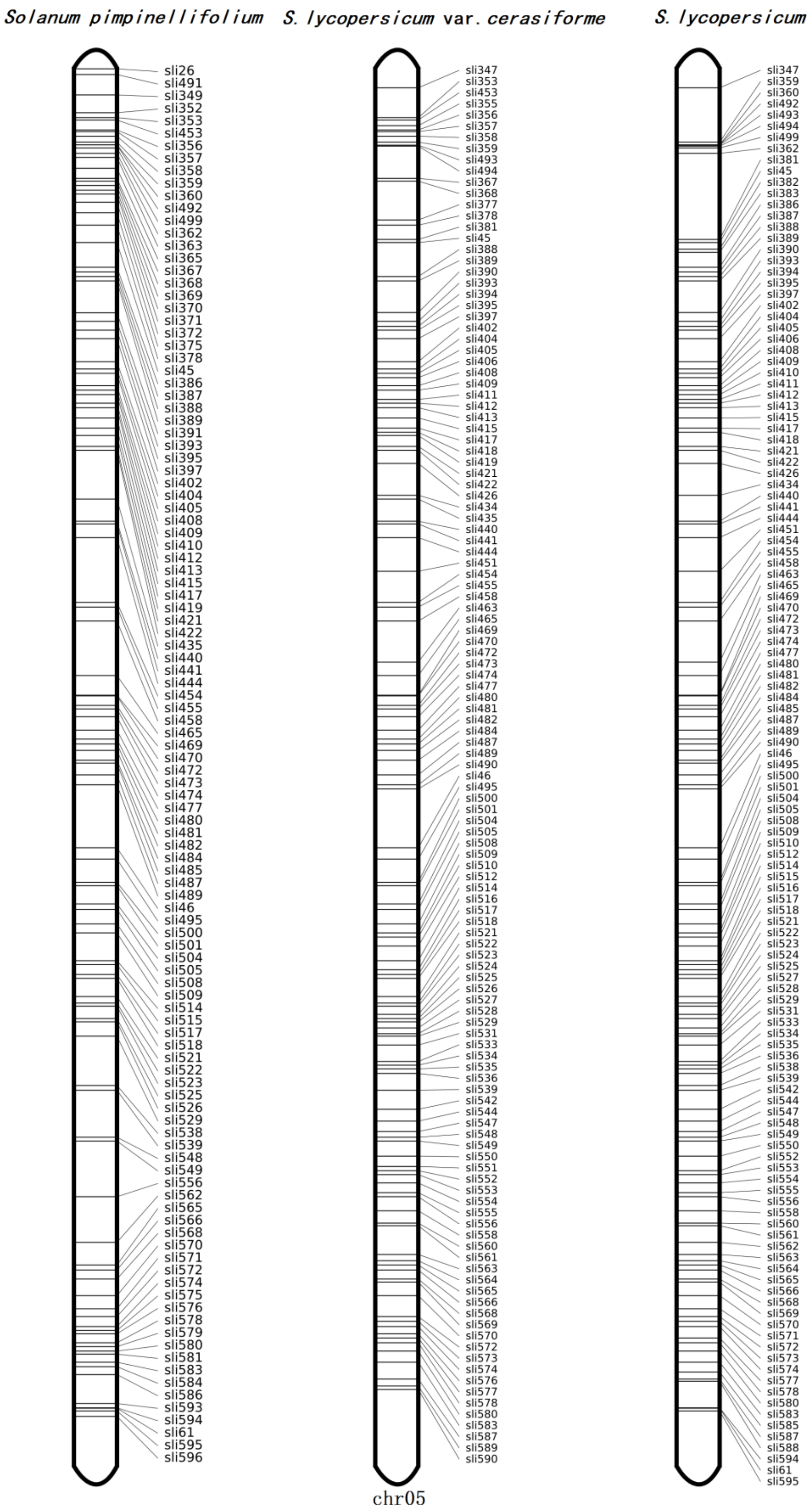
Solanum pimpinellifolium
S. lycopersicum var. cerasiforme
S. lycopersicum
sli26 sli491 sli349 sli352 sli353 sli453 sli356 sli357 sli358 sli359 sli360 sli492 sli499 sli362 sli363 sli365 sli367 sli368 sli369 sli370 sli371 sli372 sli375 sli378 sli45 sli386 sli387 sli388 sli389 sli391 sli393 sli395 sli397 sli402 sli404 sli405 sli408 sli409 sli410 sli412 sli413 sli415 sli417 sli419 sli421 sli422 sli435 sli440 sli441 sli444 sli454 sli455 sli458 sli465 sli469 sli470 sli472 sli473 sli474 sli477 sli480 sli481 sli482 sli484 sli485 sli487 sli489 sli46 sli495 sli500 sli501 sli504 sli505 sli508 sli509 sli514 sli515 sli517 sli518 sli521 sli522 sli523 sli525 sli526 sli529 sli538 sli539 sli548 sli549 sli556 sli562 sli565 sli566 sli568 sli570 sli571 sli572 sli574 sli575 sli576 sli578 sli579 sli580 sli581 sli583 sli584 sli586 sli593 sli594 sli61 sli595 sli596
sli347 sli353 sli453 sli355 sli356 sli357 sli358 sli359 sli493 sli494 sli367 sli368 sli377 sli378 sli381 sli45 sli388 sli389 sli390 sli393 sli394 sli395 sli397 sli402 sli404 sli405 sli406 sli408 sli409 sli411 sli412 sli413 sli415 sli417 sli418 sli419 sli421 sli422 sli426 sli434 sli435 sli440 sli441 sli444 sli451 sli454 sli455 sli458 sli463 sli465 sli469 sli470 sli472 sli473 sli474 sli477 sli480 sli481 sli482 sli484 sli487 sli489 sli490 sli46 sli495 sli500 sli501 sli504 sli505 sli508 sli509 sli510 sli512 sli514 sli516 sli517 sli518 sli521 sli522 sli523 sli524 sli525 sli526 sli527 sli528 sli529 sli531 sli533 sli534 sli535 sli536 sli539 sli542 sli544 sli547 sli548 sli549 sli550 sli551 sli552 sli553 sli554 sli555 sli556 sli558 sli560 sli561 sli563 sli564 sli565 sli566 sli568 sli569 sli570 sli572 sli573 sli574 sli576 sli577 sli578 sli580 sli583 sli587 sli589 sli590
sli347 sli359 sli360 sli492 sli493 sli494 sli499 sli362 sli381 sli45 sli382 sli383 sli386 sli387 sli388 sli389 sli390 sli393 sli394 sli395 sli397 sli402 sli404 sli405 sli406 sli408 sli409 sli410 sli411 sli412 sli413 sli415 sli417 sli418 sli421 sli422 sli426 sli434 sli440 sli441 sli444 sli451 sli454 sli455 sli458 sli463 sli465 sli469 sli470 sli472 sli473 sli474 sli477 sli480 sli481 sli482 sli484 sli485 sli487 sli489 sli490 sli46 sli495 sli500 sli501 sli504 sli505 sli508 sli509 sli510 sli512 sli514 sli515 sli516 sli517 sli518 sli521 sli522 sli523 sli524 sli525 sli527 sli528 sli529 sli531 sli533 sli534 sli535 sli536 sli538 sli539 sli542 sli544 sli547 sli548 sli549 sli550 sli552 sli553 sli554 sli555 sli556 sli558 sli560 sli561 sli562 sli563 sli564 sli565 sli566 sli568 sli569 sli570 sli571 sli572 sli573 sli574 sli577 sli578 sli580 sli583 sli585 sli587 sli588 sli594 sli61 sli595
chr05

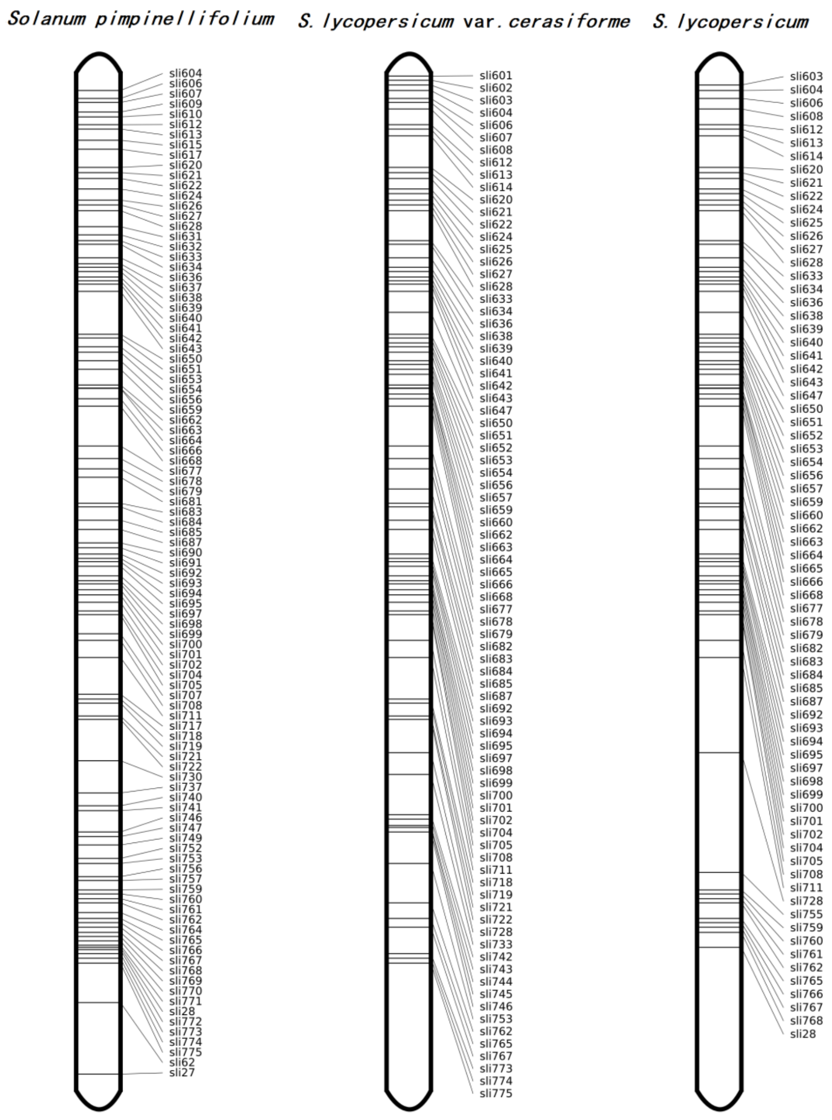
Solanum pimpinellifolium
S. lycopersicum var. cerasiforme
S. lycopersicum
sli604
sli606
sli607
sli609
sli610
sli612
sli613
sli615
sli617
sli620
sli621
sli622
sli624
sli626
sli627
sli628
sli631
sli632
sli633
sli634
sli636
sli637
sli638
sli639
sli640
sli641
sli642
sli643
sli650
sli651
sli653
sli654
sli656
sli659
sli662
sli663
sli664
sli666
sli668
sli677
sli678
sli679
sli681
sli683
sli684
sli685
sli687
sli690
sli691
sli692
sli693
sli694
sli695
sli697
sli698
sli699
sli700
sli701
sli702
sli704
sli705
sli707
sli708
sli711
sli717
sli718
sli719
sli721
sli722
sli730
sli737
sli740
sli741
sli746
sli747
sli749
sli752
sli753
sli756
sli757
sli759
sli760
sli761
sli762
sli764
sli765
sli766
sli767
sli768
sli769
sli770
sli771
sli28
sli772
sli773
sli774
sli775
sli62
sli27
sli601
sli602
sli603
sli604
sli606
sli607
sli608
sli612
sli613
sli614
sli620
sli621
sli622
sli624
sli625
sli626
sli627
sli628
sli633
sli634
sli636
sli638
sli639
sli640
sli641
sli642
sli643
sli647
sli650
sli651
sli652
sli653
sli654
sli656
sli657
sli659
sli660
sli662
sli663
sli664
sli665
sli666
sli668
sli677
sli678
sli679
sli682
sli683
sli684
sli685
sli687
sli692
sli693
sli694
sli695
sli697
sli698
sli699
sli700
sli701
sli702
sli704
sli705
sli708
sli711
sli718
sli719
sli721
sli722
sli728
sli733
sli742
sli743
sli744
sli745
sli746
sli753
sli762
sli765
sli767
sli773
sli774
sli775
sli603
sli604
sli606
sli608
sli612
sli613
sli614
sli620
sli621
sli622
sli624
sli625
sli626
sli627
sli628
sli633
sli634
sli636
sli638
sli639
sli640
sli641
sli642
sli643
sli647
sli650
sli651
sli652
sli653
sli654
sli656
sli657
sli659
sli660
sli662
sli663
sli664
sli665
sli666
sli668
sli677
sli678
sli679
sli682
sli683
sli684
sli685
sli687
sli692
sli693
sli694
sli695
sli697
sli698
sli699
sli700
sli701
sli702
sli704
sli705
sli708
sli711
sli728
sli755
sli759
sli760
sli761
sli762
sli765
sli766
sli767
sli768
sli28

chr06

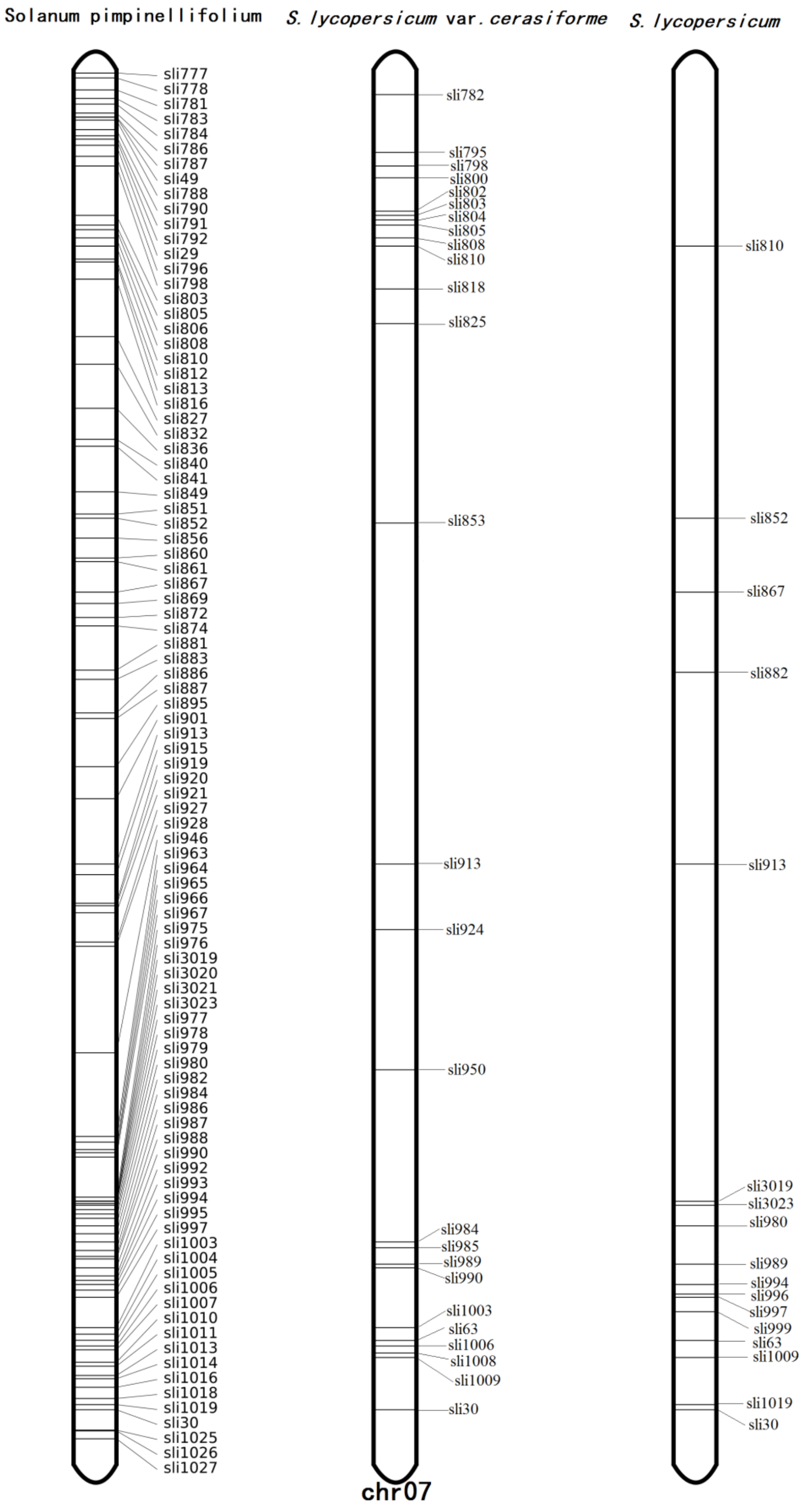

Solanum pimpinellifolium
S. lycopersicum var. cerasiforme
S. lycopersicum
sli777
sli778
sli781
sli783
sli784
sli786
sli787
sli49
sli788
sli790
sli791
sli792
sli29
sli796
sli798
sli803
sli805
sli806
sli808
sli810
sli812
sli813
sli816
sli827
sli832
sli836
sli840
sli841
sli849
sli851
sli852
sli856
sli860
sli861
sli867
sli869
sli872
sli874
sli881
sli883
sli886
sli887
sli895
sli901
sli913
sli915
sli919
sli920
sli921
sli927
sli928
sli946
sli963
sli964
sli965
sli966
sli967
sli975
sli976
sli3019
sli3020
sli3021
sli3023
sli977
sli978
sli979
sli980
sli982
sli984
sli986
sli987
sli988
sli990
sli992
sli993
sli994
sli995
sli997
sli1003
sli1004
sli1005
sli1006
sli1007
sli1010
sli1011
sli1013
sli1014
sli1016
sli1018
sli1019
sli30
sli1025
sli1026
sli1027
sli782
sli795
sli798
sli800
sli802
sli803
sli804
sli805
sli808
sli810
sli818
sli825
sli853
sli913
sli924
sli950
sli984
sli985
sli989
sli990
sli1003
sli63
sli1006
sli1008
sli1009
sli30
sli810
sli852
sli867
sli882
sli913
sli3019
sli3023
sli980
sli989
sli994
sli996
sli997
sli999
sli63
sli1009
sli1019
sli30
chr07

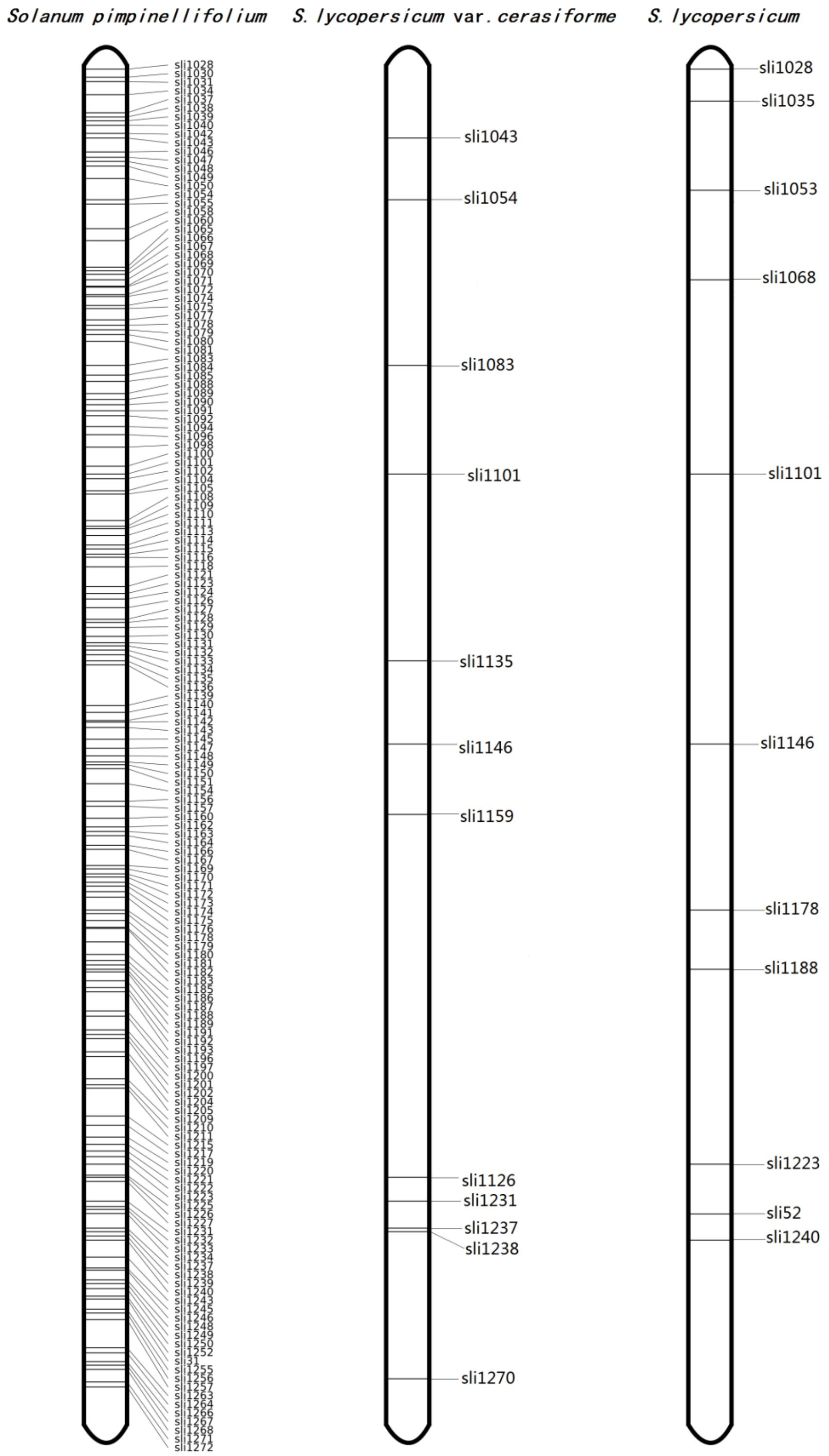
Solanum pimpinellifolium
S. lycopersicum var. cerasiforme
S. lycopersicum
sli1043
sli1054
sli1083
sli1101
sli1135
sli1146
sli1159
sli1126
sli1231
sli1237
sli1238
sli1270
sli1028
sli1035
sli1053
sli1068
sli1101
sli1146
sli1178
sli1188
sli1223
sli52
sli1240

chr08

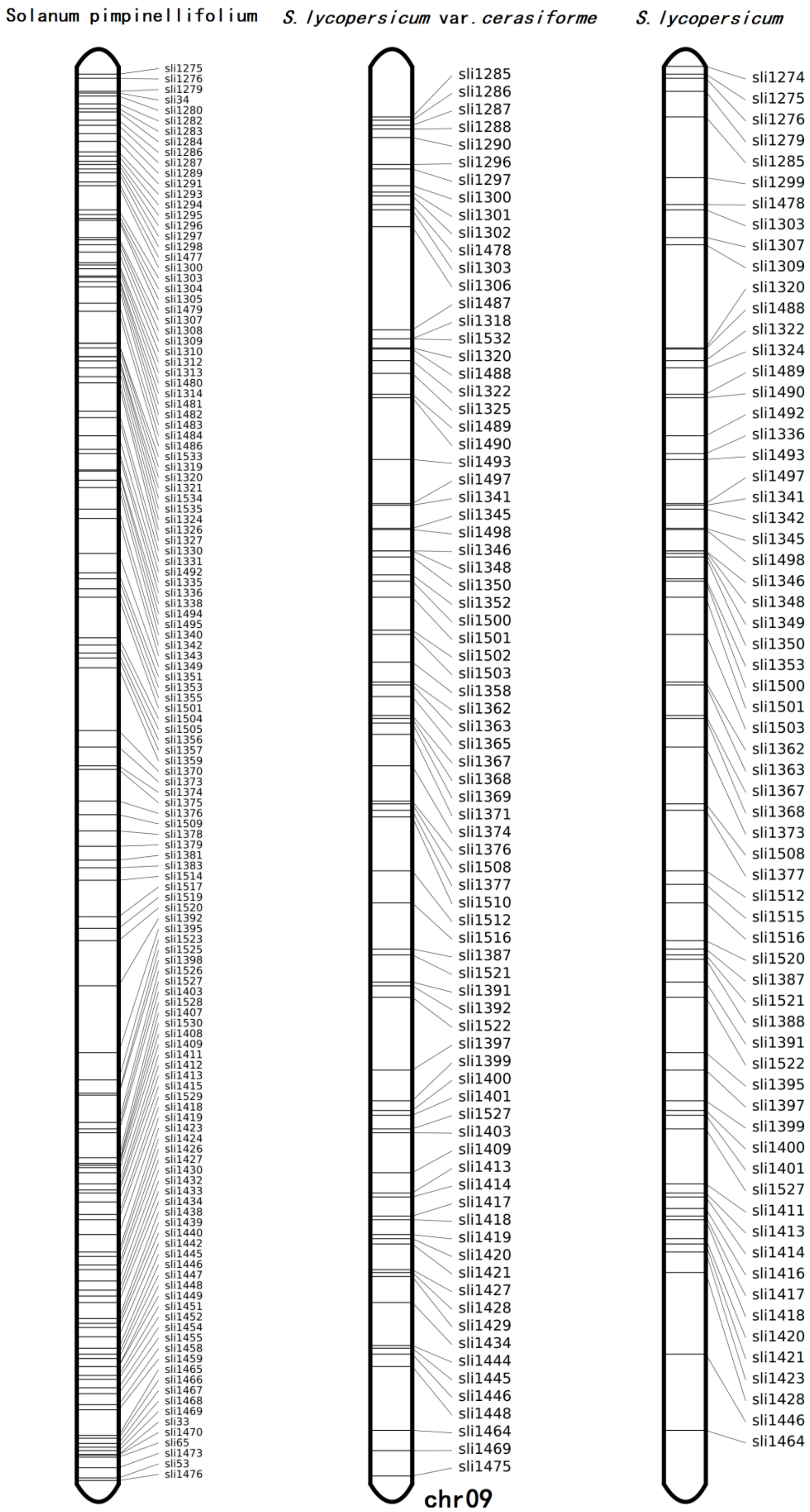
Solanum pimpinellifolium
S. lycopersicum var. cerasiforme
S. lycopersicum
sli1275
sli1276
sli1279
sli34
sli1280
sli1282
sli1283
sli1284
sli1286
sli1287
sli1289
sli1291
sli1293
sli1294
sli1295
sli1296
sli1297
sli1298
sli1477
sli1300
sli1303
sli1304
sli1305
sli1479
sli1307
sli1308
sli1309
sli1310
sli1312
sli1313
sli1480
sli1314
sli1481
sli1482
sli1483
sli1484
sli1486
sli1533
sli1319
sli1320
sli1321
sli1534
sli1535
sli1324
sli1326
sli1327
sli1330
sli1331
sli1492
sli1335
sli1336
sli1338
sli1494
sli1495
sli1340
sli1342
sli1343
sli1349
sli1351
sli1353
sli1355
sli1501
sli1504
sli1505
sli1356
sli1357
sli1359
sli1370
sli1373
sli1374
sli1375
sli1376
sli1509
sli1378
sli1379
sli1381
sli1383
sli1514
sli1517
sli1519
sli1520
sli1392
sli1395
sli1523
sli1525
sli1398
sli1526
sli1527
sli1403
sli1528
sli1407
sli1530
sli1408
sli1409
sli1411
sli1412
sli1413
sli1415
sli1529
sli1418
sli1419
sli1423
sli1424
sli1426
sli1427
sli1430
sli1432
sli1433
sli1434
sli1438
sli1439
sli1440
sli1442
sli1445
sli1446
sli1447
sli1448
sli1449
sli1451
sli1452
sli1454
sli1455
sli1458
sli1459
sli1465
sli1466
sli1467
sli1468
sli1469
sli33
sli1470
sli65
sli1473
sli53
sli1476
sli1285
sli1286
sli1287
sli1288
sli1290
sli1296
sli1297
sli1300
sli1301
sli1302
sli1478
sli1303
sli1306
sli1487
sli1318
sli1532
sli1320
sli1488
sli1322
sli1325
sli1489
sli1490
sli1493
sli1497
sli1341
sli1345
sli1498
sli1346
sli1348
sli1350
sli1352
sli1500
sli1501
sli1502
sli1503
sli1358
sli1362
sli1363
sli1365
sli1367
sli1368
sli1369
sli1371
sli1374
sli1376
sli1508
sli1377
sli1510
sli1512
sli1516
sli1387
sli1521
sli1391
sli1392
sli1522
sli1397
sli1399
sli1400
sli1401
sli1527
sli1403
sli1409
sli1413
sli1414
sli1417
sli1418
sli1419
sli1420
sli1421
sli1427
sli1428
sli1429
sli1434
sli1444
sli1445
sli1446
sli1448
sli1464
sli1469
sli1475
chr09
sli1274
sli1275
sli1276
sli1279
sli1285
sli1299
sli1478
sli1303
sli1307
sli1309
sli1320
sli1488
sli1322
sli1324
sli1489
sli1490
sli1492
sli1336
sli1493
sli1497
sli1341
sli1342
sli1345
sli1498
sli1346
sli1348
sli1349
sli1350
sli1353
sli1500
sli1501
sli1503
sli1362
sli1363
sli1367
sli1368
sli1373
sli1508
sli1377
sli1512
sli1515
sli1516
sli1520
sli1387
sli1521
sli1388
sli1391
sli1522
sli1395
sli1397
sli1399
sli1400
sli1401
sli1527
sli1411
sli1413
sli1414
sli1416
sli1417
sli1418
sli1420
sli1421
sli1423
sli1428
sli1446
sli1464

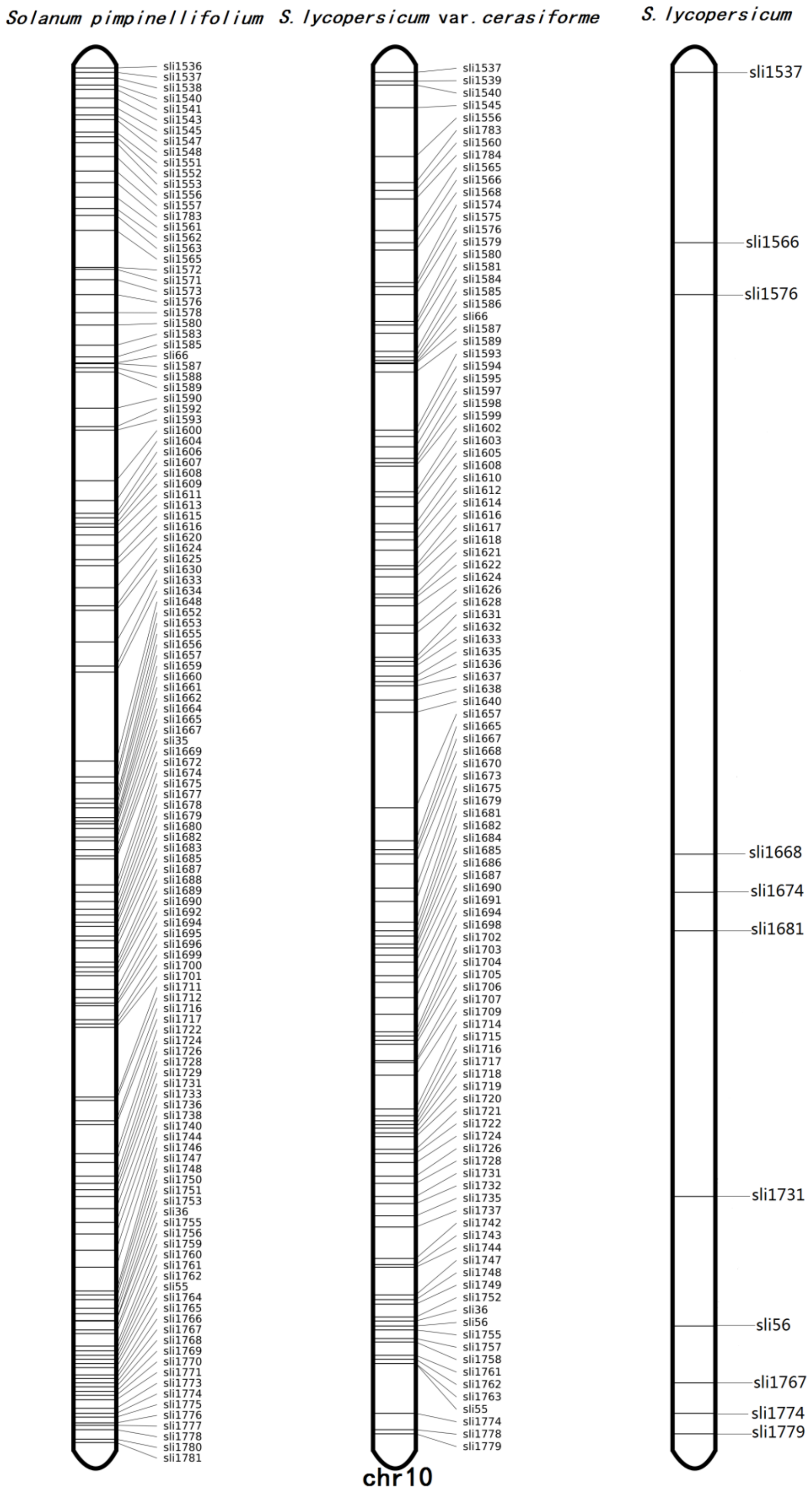
Solanum pimpinellifolium
S. lycopersicum var. cerasiforme
S. lycopersicum
sli1536
sli1537
sli1538
sli1540
sli1541
sli1543
sli1545
sli1547
sli1548
sli1551
sli1552
sli1553
sli1556
sli1557
sli1783
sli1561
sli1562
sli1563
sli1565
sli1572
sli1571
sli1573
sli1576
sli1578
sli1580
sli1583
sli1585
sli66
sli1587
sli1588
sli1589
sli1590
sli1592
sli1593
sli1600
sli1604
sli1606
sli1607
sli1608
sli1609
sli1611
sli1613
sli1615
sli1616
sli1620
sli1624
sli1625
sli1630
sli1633
sli1634
sli1648
sli1652
sli1653
sli1655
sli1656
sli1657
sli1659
sli1660
sli1661
sli1662
sli1664
sli1665
sli1667
sli35
sli1669
sli1672
sli1674
sli1675
sli1677
sli1678
sli1679
sli1680
sli1682
sli1683
sli1685
sli1687
sli1688
sli1689
sli1690
sli1692
sli1694
sli1695
sli1696
sli1699
sli1700
sli1701
sli1711
sli1712
sli1716
sli1717
sli1722
sli1724
sli1726
sli1728
sli1729
sli1731
sli1733
sli1736
sli1738
sli1740
sli1744
sli1746
sli1747
sli1748
sli1750
sli1751
sli1753
sli36
sli1755
sli1756
sli1759
sli1760
sli1761
sli1762
sli55
sli1764
sli1765
sli1766
sli1767
sli1768
sli1769
sli1770
sli1771
sli1773
sli1774
sli1775
sli1776
sli1777
sli1778
sli1780
sli1781
sli1537
sli1539
sli1540
sli1545
sli1556
sli1783
sli1560
sli1784
sli1565
sli1566
sli1568
sli1574
sli1575
sli1576
sli1579
sli1580
sli1581
sli1584
sli1585
sli1586
sli66
sli1587
sli1589
sli1593
sli1594
sli1595
sli1597
sli1598
sli1599
sli1602
sli1603
sli1605
sli1608
sli1610
sli1612
sli1614
sli1616
sli1617
sli1618
sli1621
sli1622
sli1624
sli1626
sli1628
sli1631
sli1632
sli1633
sli1635
sli1636
sli1637
sli1638
sli1640
sli1657
sli1665
sli1667
sli1668
sli1670
sli1673
sli1675
sli1679
sli1681
sli1682
sli1684
sli1685
sli1686
sli1687
sli1690
sli1691
sli1694
sli1698
sli1702
sli1703
sli1704
sli1705
sli1706
sli1707
sli1709
sli1714
sli1715
sli1716
sli1717
sli1718
sli1719
sli1720
sli1721
sli1722
sli1724
sli1726
sli1728
sli1731
sli1732
sli1735
sli1737
sli1742
sli1743
sli1744
sli1747
sli1748
sli1749
sli1752
sli36
sli56
sli1755
sli1757
sli1758
sli1761
sli1762
sli1763
sli55
sli1774
sli1778
sli1779
chr10
sli1537
sli1566
sli1576
sli1668
sli1674
sli1681
sli1731
sli56
sli1767
sli1774
sli1779

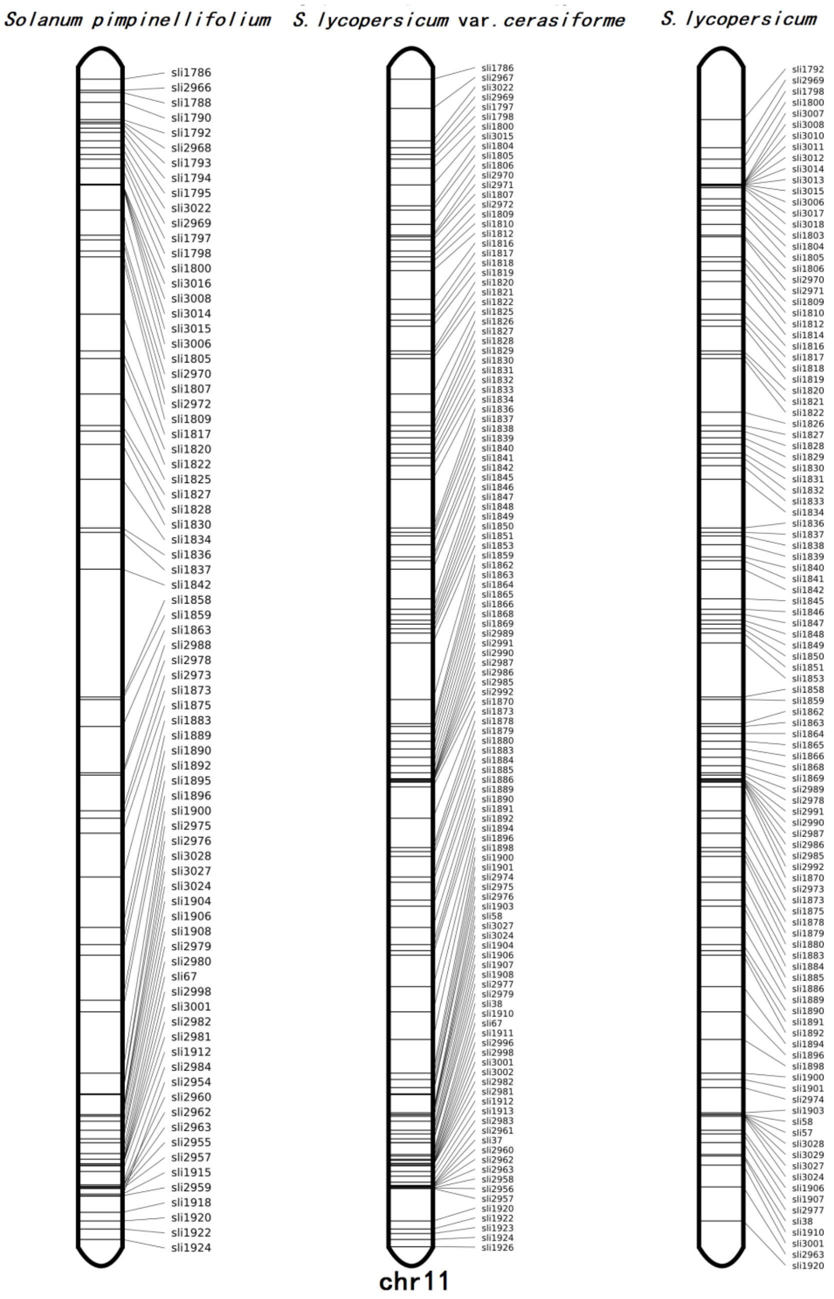

Solanum pimpinellifolium
S. lycopersicum var. cerasiforme
S. lycopersicum
sli1786
sli2966
sli1788
sli1790
sli1792
sli2968
sli1793
sli1794
sli1795
sli3022
sli2969
sli1797
sli1798
sli1800
sli3016
sli3008
sli3014
sli3015
sli3006
sli1805
sli2970
sli1807
sli2972
sli1809
sli1817
sli1820
sli1822
sli1825
sli1827
sli1828
sli1830
sli1834
sli1836
sli1837
sli1842
sli1858
sli1859
sli1863
sli2988
sli2978
sli2973
sli1873
sli1875
sli1883
sli1889
sli1890
sli1892
sli1895
sli1896
sli1900
sli2975
sli2976
sli3028
sli3027
sli3024
sli1904
sli1906
sli1908
sli2979
sli2980
sli67
sli2998
sli3001
sli2982
sli2981
sli1912
sli2984
sli2954
sli2960
sli2962
sli2963
sli2955
sli2957
sli1915
sli2959
sli1918
sli1920
sli1922
sli1924
sli1786
sli2967
sli3022
sli2969
sli1797
sli1798
sli1800
sli3015
sli1804
sli1805
sli1806
sli2970
sli2971
sli1807
sli2972
sli1809
sli1810
sli1812
sli1816
sli1817
sli1818
sli1819
sli1820
sli1821
sli1822
sli1825
sli1826
sli1827
sli1828
sli1829
sli1830
sli1831
sli1832
sli1833
sli1834
sli1836
sli1837
sli1838
sli1839
sli1840
sli1841
sli1842
sli1845
sli1846
sli1847
sli1848
sli1849
sli1850
sli1851
sli1853
sli1859
sli1862
sli1863
sli1864
sli1865
sli1866
sli1868
sli1869
sli2989
sli2991
sli2990
sli2987
sli2986
sli2985
sli2992
sli1870
sli1873
sli1878
sli1879
sli1880
sli1883
sli1884
sli1885
sli1886
sli1889
sli1890
sli1891
sli1892
sli1894
sli1896
sli1898
sli1900
sli1901
sli2974
sli2975
sli2976
sli1903
sli58
sli3027
sli3024
sli1904
sli1906
sli1907
sli1908
sli2977
sli2979
sli38
sli1910
sli67
sli1911
sli2996
sli2998
sli3001
sli3002
sli2982
sli2981
sli1912
sli1913
sli2983
sli2961
sli37
sli2960
sli2962
sli2963
sli2958
sli2956
sli2957
sli1920
sli1922
sli1923
sli1924
sli1926
chr11
sli1792
sli2969
sli1798
sli1800
sli3007
sli3008
sli3010
sli3011
sli3012
sli3014
sli3013
sli3015
sli3006
sli3017
sli3018
sli1803
sli1804
sli1805
sli1806
sli2970
sli2971
sli1809
sli1810
sli1812
sli1814
sli1816
sli1817
sli1818
sli1819
sli1820
sli1821
sli1822
sli1826
sli1827
sli1828
sli1829
sli1830
sli1831
sli1832
sli1833
sli1834
sli1836
sli1837
sli1838
sli1839
sli1840
sli1841
sli1842
sli1845
sli1846
sli1847
sli1848
sli1849
sli1850
sli1851
sli1853
sli1858
sli1859
sli1862
sli1863
sli1864
sli1865
sli1866
sli1868
sli1869
sli2989
sli2978
sli2991
sli2990
sli2987
sli2986
sli2985
sli2992
sli1870
sli2973
sli1873
sli1875
sli1878
sli1879
sli1880
sli1883
sli1884
sli1885
sli1886
sli1889
sli1890
sli1891
sli1892
sli1894
sli1896
sli1898
sli1900
sli1901
sli2974
sli1903
sli58
sli57
sli3028
sli3029
sli3027
sli3024
sli1906
sli1907
sli2977
sli38
sli1910
sli3001
sli2963
sli1920

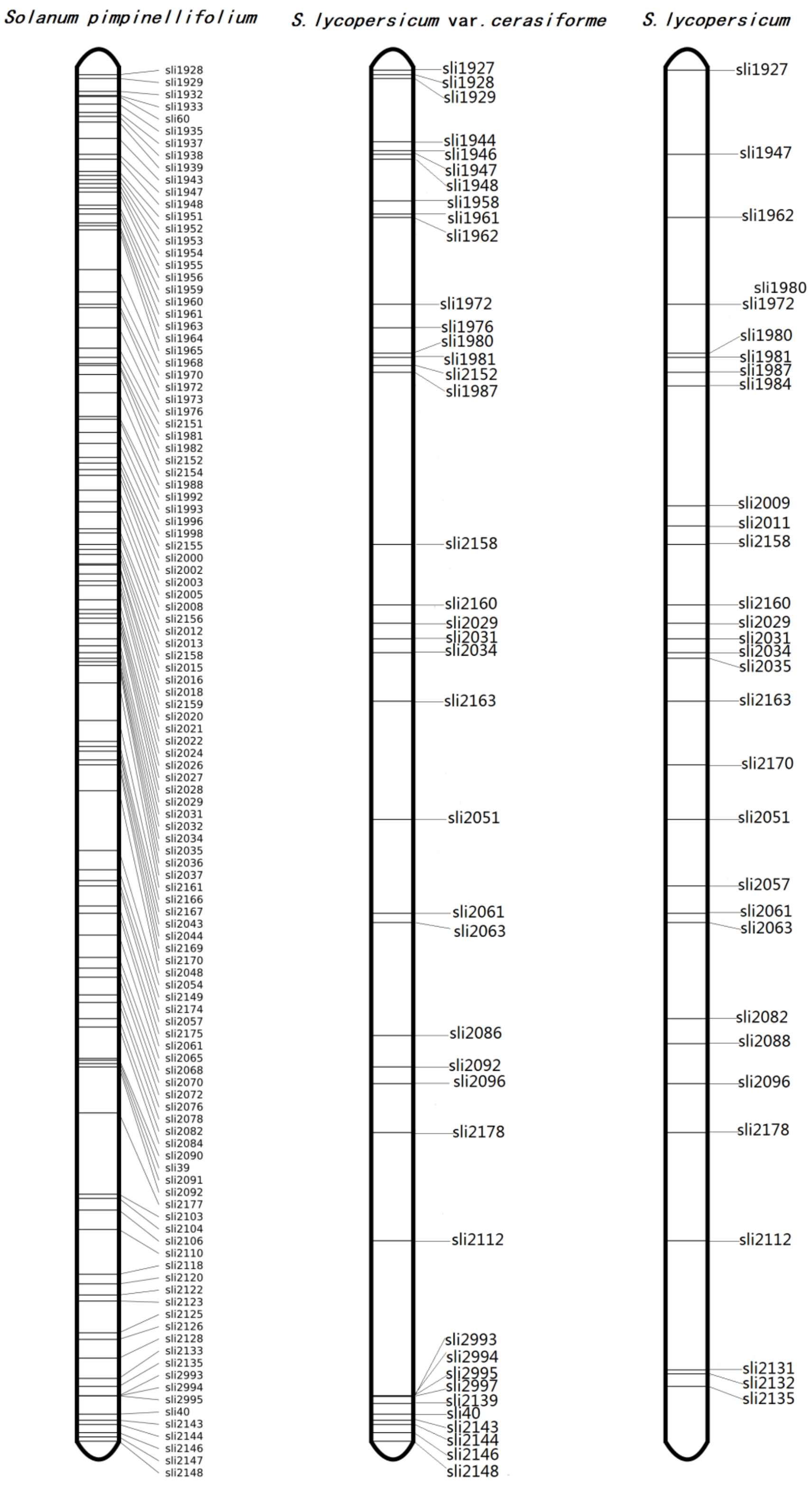
Solanum pimpinellifolium
sli1928
sli1929
sli1932
sli1933
sli60
sli1935
sli1937
sli1938
sli1939
sli1943
sli1947
sli1948
sli1951
sli1952
sli1953
sli1954
sli1955
sli1956
sli1959
sli1960
sli1961
sli1963
sli1964
sli1965
sli1968
sli1970
sli1972
sli1973
sli1976
sli2151
sli1981
sli1982
sli2152
sli2154
sli1988
sli1992
sli1993
sli1996
sli1998
sli2155
sli2000
sli2002
sli2003
sli2005
sli2008
sli2156
sli2012
sli2013
sli2158
sli2015
sli2016
sli2018
sli2159
sli2020
sli2021
sli2022
sli2024
sli2026
sli2027
sli2028
sli2029
sli2031
sli2032
sli2034
sli2035
sli2036
sli2037
sli2161
sli2166
sli2167
sli2043
sli2044
sli2169
sli2170
sli2048
sli2054
sli2149
sli2174
sli2057
sli2175
sli2061
sli2065
sli2068
sli2070
sli2072
sli2076
sli2078
sli2082
sli2084
sli2090
sli39
sli2091
sli2092
sli2177
sli2103
sli2104
sli2106
sli2110
sli2118
sli2120
sli2122
sli2123
sli2125
sli2126
sli2128
sli2133
sli2135
sli2993
sli2994
sli2995
sli40
sli2143
sli2144
sli2146
sli2147
sli2148
S. lycopersicum var. cerasiforme
sli1927
sli1928
sli1929
sli1944
sli1946
sli1947
sli1948
sli1958
sli1961
sli1962
sli1972
sli1976
sli1980
sli1981
sli2152
sli1987
sli2158
sli2160
sli2029
sli2031
sli2034
sli2163
sli2051
sli2061
sli2063
sli2086
sli2092
sli2096
sli2178
sli2112
sli2993
sli2994
sli2995
sli2997
sli2139
sli40
sli2143
sli2144
sli2146
sli2148
S. lycopersicum
sli1927
sli1947
sli1962
sli1980
sli1972
sli1980
sli1981
sli1987
sli1984
sli2009
sli2011
sli2158
sli2160
sli2029
sli2031
sli2034
sli2035
sli2163
sli2170
sli2051
sli2057
sli2061
sli2063
sli2082
sli2088
sli2096
sli2178
sli2112
sli2131
sli2132
sli2135

chr12
