# Supplementary material for: In Silico Identification and Experimental Validation of Insertion–Deletion Polymorphisms in Tomato Genome
Source: DNA Res. 2014 Mar 11;21(4):429–38. doi: 10.1093/dnares/dsu008 (PMC4131836; doi:10.1093/dnares/dsu008)
Supplement: Supplementary Data [file supp_dsu008_dsu008supp.doc]

**Supplementary Table S1**. Chromosomal position, primer sequence, length of insertion/deletion, number of alleles and polymorphic information content for 2,272 InDel markers in tomato.

**Supplementary Table S2**. Sizes and numbers of InDels predicted between LA 1589 and Heinz 1706, and percentage of non-detectable polymorphism for each InDel size

**Supplementary Table S3**. Pairwise comparison of number and percentage (in parentheses) of InDels among 22 tomato genotypes

**Supplementary Table S4**. Genes with InDels in coding region

**Supplementary Figure S1.** Physical map of InDels on tomato chromosomes. The relative map position of each InDel can be found in Supplementary Table S1.

**Supplementary Figure S2.** Physical map of polymorphic InDels on 12 chromosomes in *S. pimpinellifolium*, *S. lycopersicum* var. *cerasiforme*, and *S. lycopersicum***.** The relative map position of each InDel can be found in Supplementary Table S1.
