# Supplementary figures and images for: In Silico Identification and Experimental Validation of Insertion–Deletion Polymorphisms in Tomato Genome
Source: DNA Res. 2014 Mar 11;21(4):429–38. doi: 10.1093/dnares/dsu008 (PMC4131836; doi:10.1093/dnares/dsu008)

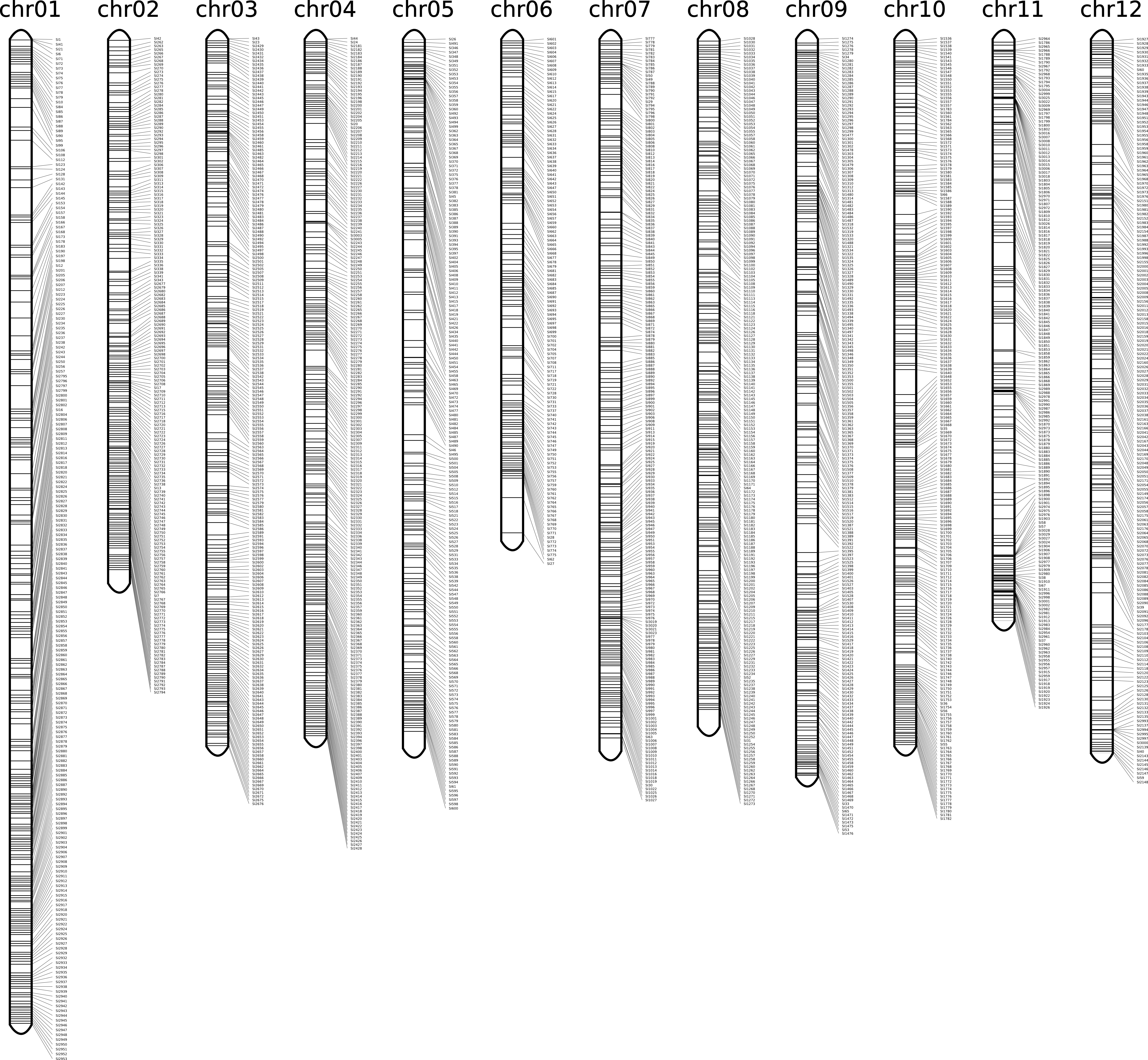

Supplement: Supplementary Data [file supp_dsu008_dsu008supp_fig1.tif]
